# HIV drug resistance prediction with weighted categorical kernel functions

**DOI:** 10.1186/s12859-019-2991-2

**Published:** 2019-07-30

**Authors:** Elies Ramon, Lluís Belanche-Muñoz, Miguel Pérez-Enciso

**Affiliations:** 1 Centre for Research in Agricultural Genomics (CRAG), CSIC-IRTA-UAB-UB Consortium, Campus UAB, 08193 Bellaterra, Barcelona, Spain; 2 Computer Science Department, Technical University of Catalonia, Carrer de Jordi Girona 1-3, 08034 Barcelona, Spain; 3 Institució Catalana de Recerca i Estudis Avançats (ICREA), Passeig de Lluís Companys 23, 08010 Barcelona, Spain

**Keywords:** HIV, Drug resistance prediction, Categorical kernel, Weighted kernel, PI, NRTI, NNRTI, INI, Machine learning, Support vector machine, Random Forest, Kernel PCA

## Abstract

**Background:**

Antiretroviral drugs are a very effective therapy against HIV infection. However, the high mutation rate of HIV permits the emergence of variants that can be resistant to the drug treatment. Predicting drug resistance to previously unobserved variants is therefore very important for an optimum medical treatment. In this paper, we propose the use of weighted categorical kernel functions to predict drug resistance from virus sequence data. These kernel functions are very simple to implement and are able to take into account HIV data particularities, such as allele mixtures, and to weigh the different importance of each protein residue, as it is known that not all positions contribute equally to the resistance.

**Results:**

We analyzed 21 drugs of four classes: protease inhibitors (PI), integrase inhibitors (INI), nucleoside reverse transcriptase inhibitors (NRTI) and non-nucleoside reverse transcriptase inhibitors (NNRTI). We compared two categorical kernel functions, Overlap and Jaccard, against two well-known noncategorical kernel functions (Linear and RBF) and Random Forest (RF). Weighted versions of these kernels were also considered, where the weights were obtained from the RF decrease in node impurity. The Jaccard kernel was the best method, either in its weighted or unweighted form, for 20 out of the 21 drugs.

**Conclusions:**

Results show that kernels that take into account both the categorical nature of the data and the presence of mixtures consistently result in the best prediction model. The advantage of including weights depended on the protein targeted by the drug. In the case of reverse transcriptase, weights based in the relative importance of each position clearly increased the prediction performance, while the improvement in the protease was much smaller. This seems to be related to the distribution of weights, as measured by the Gini index. All methods described, together with documentation and examples, are freely available at https://bitbucket.org/elies_ramon/catkern.

**Electronic supplementary material:**

The online version of this article (10.1186/s12859-019-2991-2) contains supplementary material, which is available to authorized users.

## Background

HIV is a retrovirus that infects human immune cells, causing a progressive weakening of the immune system. When untreated, the affected person develops acquired immunodeficiency syndrome (AIDS), which leads to a rise of opportunistic infections and, finally, death. HIV has infected more than 35 million people worldwide and is considered a global pandemic [1]. Despite the efforts, to date there is no definitive cure that eradicates the virus from the organism. However, the lifespan and quality of life of many people that live with HIV have expanded greatly thanks to antiretroviral therapy. Antiretroviral drugs lower the virus level in blood by targeting different stages of the virus life cycle. The most important classes of antiretroviral drugs are protease inhibitors (PIs), which target the protease, and nucleoside and non-nucleoside reverse transcriptase inhibitors (NRTIs and NNRTIs, respectively) which target the reverse transcriptase. Other classes of antiretroviral drugs are the integrase inhibitors (INIs) and the fusion inhibitors.

Some of the main reasons why HIV is so difficult to fight are its short life cycle (1–2 days), high replication rate (10^8^–10^9^ new virions each day), and high mutation rate (10^− 4^–10^− 5^ mutations per nucleotide site per replication cycle) caused because reverse transcriptase lacks proofreading activity. This permits the fast emergence of new HIV variants, some of which may be resistant to the drug treatment [2]. These variants can be transmitted, and some studies show that ~ 10% of patients who had never been on antiretroviral therapy carry at least one resistant HIV [3]. Cross-resistance (simultaneous resistance to two or more drugs, often of the same class) is also a common phenomenon. It is therefore advisable to do a resistance test before the treatment to find the best drug choice [2, 4], especially in developing countries, as recommended by the WHO and the International AIDS Society-USA Panel [3]. A resistance test can be performed in vitro, obtaining HIV samples from the patient and using them to infect host cells cultured in presence of increasing levels of drug concentration. The virus susceptibility is then obtained empirically as the IC50 [4] and usually delivered as the relative IC50 (resistance of the virus variant compared to the wild type). Another strategy is to infer the HIV variant resistance from its sequence. This can be either gene sequence or the translated protein sequence; this latter approach eliminates the noise of synonymous mutations. In any case, as genome sequencing is cheaper, faster and more widely available than performing an in vitro drug susceptibility test, much effort has been invested in developing algorithms that predict the drug resistance from the virus sequence [5].

The first attempts of automatic prediction can be traced back, at least, to the early 2000s [6]. These approaches were rule-based: study the mutational profile of the HIV variant to look for known major drug-associated resistance mutations (lists of these mutations are periodically updated and can be found in reviews, e.g., [7]). The rule-based algorithms continue to be used to this day because of their interpretability. Some publicly available examples are the Stanford HIVdb, Rega or ANRS softwares [5]. However, the aforementioned high mutation rate of the HIV, which favors the emergence of large numbers of new resistance mutations and complex mutational patterns, makes the rule-based approach suboptimal. In this scenario machine learning methods can be extremely helpful, especially in recent years with the increasing size of available data. This second approach is also very popular and there exists machine learning software to predict resistance online [8, 9]. Different methods have been proposed, the most common ones being Linear Regression [10, 11], Artificial Neural Networks (ANN) [10, 12–14], Support Vector Machines (SVMs) [10, 15, 16], Decision Trees (DT) [10, 17] and their ensemble counterpart, Random Forests (RF) [15, 16, 18, 19]. Some machine learning studies have complemented the sequence data with structural information, e.g., [11, 15, 16, 18], or have benefited from the knowledge about major drug associated mutations to perform feature selection. The inclusion of cross-resistance information in the form of ensemble methods has also been reported to improve resistance prediction [20–22].

Nevertheless, HIV sequence data specificities pose significant challenges to resistance prediction. First, sequence data is categorical in nature. However, most machine learning algorithms are designed to cope with numeric data (DT and RF being exceptions), thus obliging to perform some kind of pre-processing. A typical approach is to recode each position into *m* or *m − 1* “dummy variables”, which can take the values 0 or 1 [5]. Usually, *m* is the number of all possible alleles that can be potentially found in a position (i.e., *m* = 20 in protein sequences). However, some authors restrict the dummy variables to the drug associated mutations already appearing in the literature [6, 10, 12]. A very different approach is found in [14], where each amino acid was codified as an integer ranging 1–22 (the 20 canonical amino acids plus two extra characters B and Z). Other encodings have been used with HIV sequence data, like amino acid composition frequencies, reduced amino acid alphabets or physicochemical properties [5, 16, 20].

Another challenge is the presence of mixtures of alleles (normally two, rarely three or four) in at least one position of the viral sequence for most clinical samples. In the case of HIV, this event indicates that the patient carries two or more virus variants [4]. It is well established that HIV tends to generate viral swarms of closely related viruses (quasispecies), as a consequence of its high mutation rate [2]. Mixtures introduce ambiguity in the genotype-phenotype correlation [6] and a problem of technical nature: the vast majority of machine learning methods are not able to deal directly with these “multiallelic” codes. To our knowledge, algorithms so far have handled allele mixtures with some sort of previous pre-processing of the data, e.g., keeping only the most frequent amino acid of the mixture [19], replacing the positions by a missing value [17], excluding the affected sequences [15] or expanding the data to obtain all the possible sequences that could be generated with the observed mixtures [11, 14, 18].

In this paper, we propose the use of kernel functions specifically adapted to the aforementioned HIV data intricacies, and able to integrate the relevance of the major resistance associated protein residues. Kernels are mathematical functions with interesting properties. They can be coupled to numerous machine learning algorithms, the so-called kernel methods, and provide a framework to deal with data of virtually any type (e.g. vectors, strings, graphs). They can also encode complementary knowledge about a problem, as long as some mathematical conditions are satisfied [23]. Our aim using kernel functions that address the aforementioned HIV data particularities was not only to improve prediction, but also reduce pre-processing, thus preserving the data integrity and lowering the risk of inserting spurious patterns.

## Methods

### Datasets and data pre-processing

The Genotype-Phenotype Stanford HIV Drug Resistance Database [24] is a public dataset with sequences from HIV isolates and its relative susceptibility to several antiretroviral drugs. We retrieved the PhenoSense dataset from Stanford webpage (version date: 2019-2-20). The data is split in four databases (PI, NRTI, NNRTI and INI), which contain between 1,000–3,500 HIV isolates. INI is a new addition to the Stanford database and includes some of the drugs most recently approved for therapeutic use. The complete dataset contains eight protease inhibitors: atazanavir (ATV), darunavir (DRV), fosamprenavir (FPV), indinavir (IDV), lopinavir (LPV), nelfinavir (NFV), saquinavir (SQV) and tipranavir (TPV); five integrase inhibitors: bictegravir (BIC), cabotegravir (CAB), dolutegravir (DTG), elvitegravir (EVG) and raltegravir (RAL); and two classes of reverse transcriptase inhibitors: six NRTIs, lamivudine (3TC), abacavir (ABC), zidovudine (AZT), stavudine (D4T), didanosine (DDI) and tenofovir (TDF); and four NNRTIs, efavirenz (EFV), etravirine (ETR), nevirapine (NVP) and rilpivirine (RPV). Sequence length is 99 amino acids in the case of PI database, 288 in the case of INI database and 240 in the case of NRTI and NNRTI databases. The dataset contains the strain virus resistance (relative IC50) to each drug, and the sequence of the protein targeted by this drug. We built the regression models for each drug separately, taking each polymorphic protein position as a predictor variable and the drug resistance value as the target variable. Since the distributions of resistances are highly skewed we used the log-transformed values, as recommended in [5]. Redundant viruses obtained from the same patient were removed to minimize bias. We deleted all sequences affected by events that changed protein length (protein truncations, insertions and deletions). These events were uncommon in the dataset and affected less than 5% of HIV sequences. Also, we removed all isolates with one or more missing values. Missing values are present in the target variables as well as in the sequences, because not all HIV isolates have been tested for all drugs. The final number of data instances for each drug is shown in Table 1. To ensure a minimum of data rows for training/test partitions and cross-validation, we did not consider drugs with a sample size lower than 100.

**Table 1:** Final number of HIV isolates per drug

Drug	Data size	Drug	Data size	Drug	Data size	Drug	Data size
ATV	1058	SQV	1603	DDI	1511	BIC	84
DRV	665	TPV	766	TDF	1236	CAB	0
FPV	1559	3TC	1482	EFV	1511	DTG	209
IDV	1607	ABC	1513	ETR	502	EVG	577
LPV	1372	AZT	1502	NVP	1517	RAL	636
NFV	1654	D4T	1510	RPV	181		

### Methods

We compared the performance of a nonlinear, nonkernel method (RF) to a kernel method: SVMs. SVMs can be either linear or nonlinear, depending on the kernel used. The linear kernel is the simplest of all kernel functions, given by the inner product of two vectors in input space, **x** and **y**:

1 $$ {k}_{Lin}\left(\mathbf{x},\mathbf{y}\right)={\mathbf{x}}^{\mathrm{T}}\mathbf{y} $$

In our case, **x** and **y** represent the protein sequence of two HIV isolates, recoded as dummy variables [25]. We used this kernel as the linear method of reference. An alternative expression is:

2 $$ {k}_{Lin}\left(\mathbf{x},\mathbf{y}\right)=\sum \limits_{i=1}^d{w}_i{x}_i{y}_i $$

where *d* is the length of the sequence. This expression stresses the possibility of assigning a weight *w*_*i*_ to each protein position, as it is known that not all positions contribute equally to the virus resistance [2]. Weights are nonnegative and sum to one. We considered two options: the simplest one was to consider that all positions have the same importance, i.e., assigning equal weight *1/d* to all variables. The second one was including additional information into the kernels, using RF mean decrease in node impurity as a metric for position importance.

#### RBF kernel

It is a nonlinear kernel, usually defined as:

3 $$ {k}_{RBF}\left(\mathbf{x},\mathbf{y}\right)={e}^{-\gamma {\left|\left|\mathbf{x}-\mathbf{y}\right|\right|}^2} $$

Where ||**x** − **y**||^2^ is the squared Euclidean distance between two vectors, and *γ* > 0 is a hyperparameter. As in the case of the linear kernel, the original data was recoded. We also introduced the possibility of weighting the positions:

4 $$ {k}_{RBF}\left(\mathbf{x},\mathbf{y}\right)={e}^{-\gamma \sum \limits_{i=1}^d{w}_i{\left({x}_i-{y}_i\right)}^2} $$

The RBF kernel is a widely accepted default method [23, 25], so we used it as a benchmark to compare with the categorical kernels.

#### Overlap kernel

This is the most basic categorical kernel. This kernel assigns 1 if the two instances compared are equal and 0 otherwise.

5 $$ {k}_{Ov}\left({x}_i,{y}_i\right)=\left\{\begin{array}{c}1\  if\ {x}_i={y}_i\\ {}0\  if\ {x}_i\ne {y}_i\end{array}\right. $$

where *x*_*i*_ and *y*_*i*_ represent the alleles of a given protein position *i* in two HIV sequences, **x** and **y**.

#### Jaccard kernel

The Jaccard index measures the similarity between two finite sets and is a valid kernel function [26]. We used it to handle allele mixtures, while in the rest of methods we randomly sampled one allele of the mixture. Letting again *i* denote a given protein position (so that *X*_*i*_ and *Y*_*i*_ are non-empty sets of alleles in the *i*-th position for isolates **x** and **y**) then:

6 $$ {k}_{Jac}\left({X}_i,{Y}_i\right)=\frac{\mid {X}_i\cap {Y}_i\mid }{\mid {X}_i\cup {Y}_i\mid } $$

When ∣*X*_*i*_ ∣  =  ∣ *Y*_*i*_ ∣  = 1, i.e., none of the individuals have an allele mixture at that i-th position, Jaccard reduces to the Overlap kernel. Unlike Overlap, the Jaccard kernel can deal simultaneously with allele mixtures and categorical data.

#### “RBF-like” categorical kernels

For the whole protein sequences, we can aggregate all single position Overlap and Jaccard evaluations as the convex combination of kernels evaluations (Eq. 5 or 6) and position weights. This results in a valid kernel function, since the product of a positive scalar and a kernel is a kernel, and the sum of kernels is also a kernel. To ensure that the only difference between categorical kernels and RBF was the categorical part, we introduced an exponential factor and the hyperparameter *γ*, in a way analogous to (3) and (4):

7 $$ {k}_{cat}\left(\mathrm{x},\mathrm{y}\right)={e}^{-\gamma }{e}^{\gamma \sum \limits_{i=1}^d{w}_i\cdotp k\left({x}_i,{y}_i\right)} $$

This is also a valid kernel function, since the exponential of a kernel gives another kernel, and where *e*^−*γ*^ normalizes the kernel matrix, keeping the evaluations between 0 and 1. The final versions of the Overlap and the Jaccard kernels are obtained replacing the *k*(*x*_*i*_, *y*_*i*_) term by (5) or (6), respectively. In our analyses, we compared weighted and unweighted versions for all linear, RBF, Overlap and Jaccard kernels. Thus we can ensure a fair comparison between the categorical and the noncategorical kernels.

### Stacked models

So far, we have built prediction models for each inhibitor separately. As mentioned in the Introduction, it is reported that there exists some degree of relationship between the resistance of different drugs (e.g. in case of cross-resistance). To check whether the use of this information can improve prediction, we implemented the stacking algorithm described in [22] for continuous outcomes. This meta-learner approach consists of two principal steps. In the first step, single drug models are built from the training data as usual. In the second step, the fitted values (i.e. predictions of the training data) of all drugs obtained in step 1 are used as input to a new (stacked) model, being each drug a different predictor. The method that integrates the single drug models in step 2 and delivers the definitive predictions is called a combiner algorithm. Data size largely varied between drugs (see Table 1), even within the same drug class, so we chose Decision Trees (DT) as our combiner algorithm, as they can easily handle missing data. We combined the drugs within the same database (PI, NRTI, NNRTI and INI) and applied this stacking methodology to our previously proposed weighted kernels (Linear, RBF, Overlap and Jaccard).

### Experimental setup and model tuning

To assess the performance of the methods used, each database was split at random in two partitions: training set (60% of the database) and test set (40%). Hyperparameter optimization was done by a 10 × 10 cross-validation on the training set. Once the optimum hyperparameter was found, the final model was built using the whole training set. To assess the model performance, the NMSE (Normalized Mean Square Error) between the actual and the predicted drug resistances of the test set was computed:

8 $$ NMSE\left( observed, predicted\right)=\frac{\sum {\left( observed- predicted\right)}^2}{\left(N-1\right)\cdotp \mathit{\operatorname{var}}(observed)} $$

NMSE can be understood as the fraction of target variance not explained by the model.

We repeated the whole process 40 times, each time with different 60/40 randomly split training/test partitions, to obtain an error distribution. Kernel position weights were calculated using the training set only. Note that only the Jaccard kernel can directly handle allele mixtures; for the rest of kernels and the RF, we generated 40 versions of the database randomly sampling one allele at a time. Then, the 40 replicates were used to compute all the models except Jaccard, which could deal directly with the database without further preprocessing. This way we can ensure an honest comparison between Jaccard and the rest of kernels and methods.

All analyses were implemented in the R statistical computing language [27]. A documented package implementing these methods is available at https://bitbucket.org/elies_ramon/catkern/.

### Visualization

Kernel PCA is a kernel method obtained by coupling kernel functions to a Principal Components Analysis. We used the Jaccard kernel PCA to visually check whether sequences that are considered more similar by the kernel function are also similar in their drug resistance. As this method is for visualization purposes only, we did not separate training and test sequences. Thus, we used the mean kernel weights of the 40 training sets to compute the weighted Jaccard.

To check whether the important protein positions (i.e. kernel weights) detected by RF could have an structural relevance, we highlighted our top ranking positions on the tridimensional structure of the protein. Pictures of protein-drug complexes were generated with Molsoft ICM-Browser v.3.7–2 using structural data obtained from RCSB Protein Data Bank.

### Performance comparison to other approaches

We compared our SVM plus weighted Jaccard with the ANN approach described in [14], which to our knowledge achieves the best performance so far in this dataset. We used the R interface to keras to implement the ANN. First, we followed the specifications described in [14] about the range of candidate architectures (1–3 hidden layers, with 2–10 nodes per layer, for all drugs), number of epochs and early stopping. As our dataset version and data pre-processing differ from [14], we also evaluated a different range of hyperparameters: three fixed ANN architectures (one hidden layer with 30 nodes, two hidden layers with 20 and 10 nodes respectively, and three hidden layers with 30, 20 and 10 nodes) with the L2 regularization parameter λ. Both approaches (from now on referred to as ANN1 and ANN2) were trained and tested as for the rest of methods (see: Data and dataset pre-processing), with the previously described 40 replicates, allele mixture treatment, training/test ratio and 10 × 10 cross-validation to choose the best number of layers and nodes per layer (in the case of ANN1) or λ (in the case of ANN2). We chose the best architecture obtained in training within ANN1 and ANN2 options for each drug.

## Results

As expected, HIV protein sequences showed a large variability. As many as 93% of the protease positions were polymorphic and, among these, the number of different observed alleles varied between 2 and 16. In the case of reverse transcriptase, 89% of the positions were polymorphic and the number of alleles per polymorphic position ranged between 2 and 14. Integrase was the least variable protein: 75% of the positions were polymorphic and, in these positions, the number of alleles ranged between 2 and 8. Almost 60% of the sequences had at least one allele mixture.

Figure 1 shows the NMSE distribution boxplot for four representative drugs: FPV (PI database, panel a), DDI (NRTI database, panel b), NVP (NNRTI database, panel c) and EVG (INI database, panel d). The remaining 17 boxplots can be found at Additional file 1: Figures S1-S17.

**Fig. 1:**
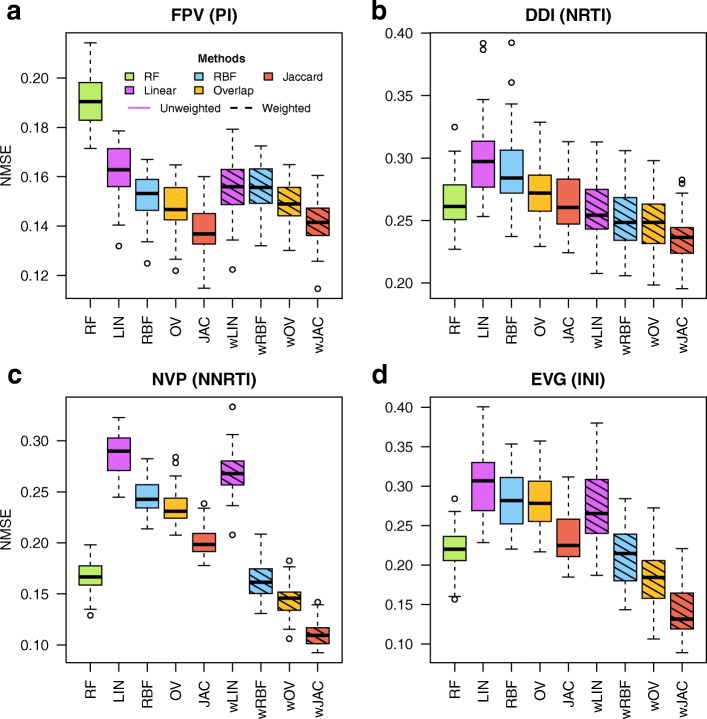
NMSE distributions for a PI (FPV, panel **a**), an NRTI (DDI, panel **b**), an NNRTI (NVP, panel **c**) and an INI (EVG, panel **d**). Note that NMSE scale varies between panels

### Performance overview

NMSE varied greatly across drugs and methods. The best prediction was achieved for 3TC, with an average NMSE ranging 0.07–0.16 depending on the method used (Additional file 1: Figure S8). The drug with worst prediction error was DTG, with an average NMSE ranging 0.65–0.75 (Additional file 1: Figure S16). This was also the second drug with lowest data size (Table 1). Not unexpectedly, methods applied to drugs with low N had considerably worse performance overall (especially DTG, RPV, ETR and TPV, but also TDF and to some extent DRV). In the PI database, errors were fairly similar across all drugs and around 0.12–0.20 on average (e.g. Figure 1a), with the sole exception of TPV, with an average NMSE ranging 0.30–0.45. In turn, predictive performances for the integrase and reverse transcriptase inhibitors were far more variable across drugs. Overall, the best method was the SVM with the Jaccard kernel (either in its weighted or in its unweighted version), which achieved the best performance in 20 out of 21 drugs.

#### Unweighted case

Nonlinear kernels performed much better than the linear kernel in almost all drugs, with the only exception of ETR and D4T. Categorical kernels outperformed RBF, although RBF was close to Overlap (or even marginally better) in some cases. Among categorical kernels, the Jaccard kernel performed better than Overlap in all inhibitors, sometimes by a large margin, as in the cases of SQV, 3TC, AZT, EFV, NVP, RAL or EVG (Fig. 1 c and d). Predictive performances of unweighted kernels and of RF were markedly different in protease with respect to integrase and transcriptase inhibitors. RF was consistently worse than kernel methods for the PI database (e.g. Figure 1a), whereas RF performance was comparable or better than those of kernel methods in both reverse transcriptase and integrase inhibitors (e.g. Figure 1b, c and d).

#### Weighted case

**Fig. 2:**
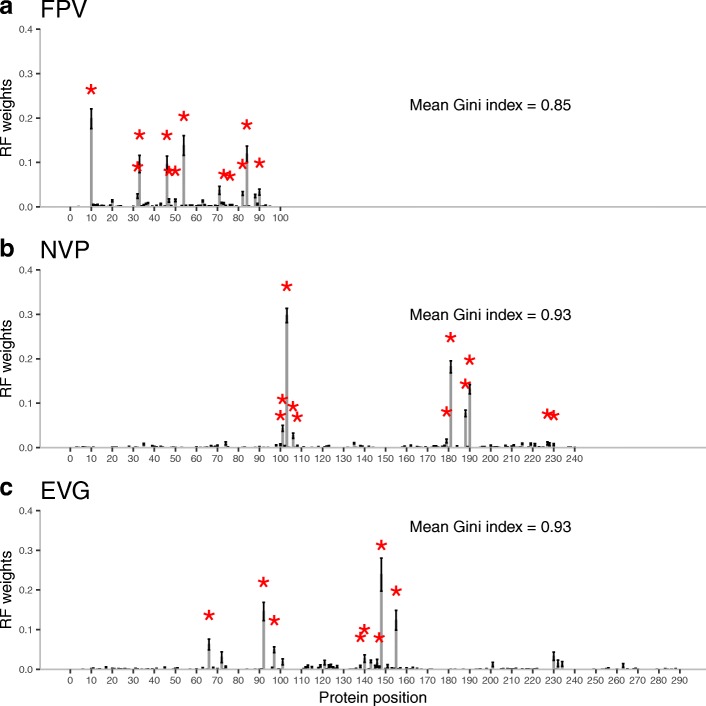
RF relative importance of each protein position for three drugs: a protease inhibitor (**a**), a reverse transcriptase inhibitor (**b**) and an integrase inhibitor (**c**). Standard error across the 40 replicates is marked with error bars. Asterisks highlight the major drug related positions reported in the literature [2]

**Fig. 3:**
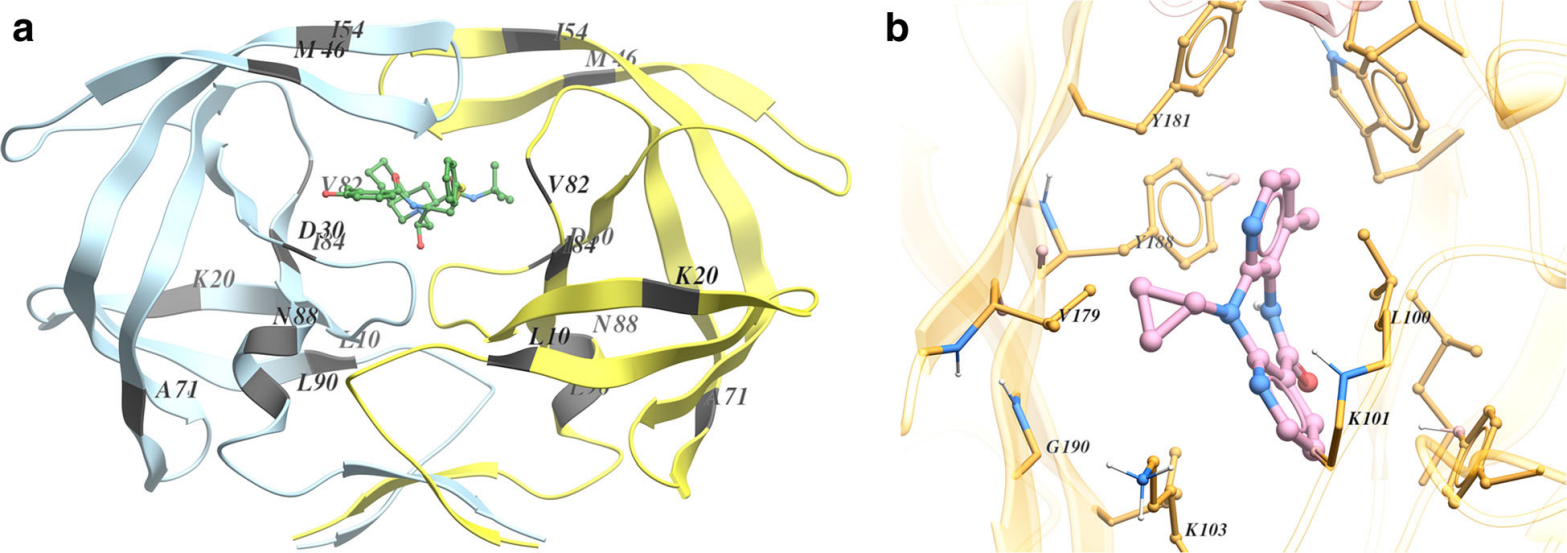
**a** Wild type protease (in yellow and blue) with an inhibitor (NFV, in green) (PDB code: 3EKX). We highlight the ten most important positions according to RF: 10, 90, 54, 46, 71, 88, 84, 30, 20 and 82. These positions are scattered throughout the protein and only a few belong to the drug binding site (e.g. 30, 82 and 84). Mutations at the binding site reduce the affinity for the inhibitor, but can impair the protease catalytic activity as a collateral damage. Mutations in distant residues are typically concurrent with these binding site mutations and often have a compensatory role (e.g. stabilizing the protease structure or restoring the catalytic activity). Position 30 appears to be important only in the case of the NFV drug, while the other positions are found in all (or almost all) protease inhibitors. This agrees with the literature [2]. **b** Binding pocket of the reverse transcriptase (in yellow) with an NNRTI (NVP, in pink) (PDB code: 3V81). We highlight the five most important positions for NVP according to RF: 103, 181, 190, 188 and 101. All these positions reside in the NNRTI binding pocket of the enzyme, and also appear in the other NNRTIs analyzed. Thus, in EFV, we find 100 (but not 181) in the top 5; and in ETR, we have 179 instead of 188 (also highlighted). Positions 103 and 101 are located near the entry of the inhibitor binding pocket and, when mutated, interfere with the entrance of the inhibitor to the binding site. Y181 and Y188 have a crucial contribution the NVP binding via stacking interactions between its side chains and the inhibitor aromatic groups. G190 mutations lead to resistance through steric hindrance, because of the substitution by a more voluminous side chain. L100 effect is also related to steric hindrance [2]

Figure 2 shows three representative examples of the weights obtained from RF. The remaining plots are shown in Additional file 2: Figures S18-S35. We ascertained that RF detected most of the major resistance associated positions described in the literature (e.g. review in [2]). Overall, a higher percentage of relevant positions were identified in protease inhibitors than in both reverse transcriptase and integrase inhibitors. To evaluate this numerically, we computed the Gini index of the RF importance distributions for each of the drugs. This index is shown in Fig. 2 and Additional file 2. We also noticed differences regarding the location of the important positions in the tridimensional structures of protease (Fig. 3a) and reverse transcriptase (Fig. 3b). The most important protease positions according to RF are distributed over the whole structure, whereas in the case of the reverse transcriptase they are located at the drug binding site.

As for predictive performance, weighting was more effective in integrase and reverse transcriptase inhibitors than in protease inhibitors. In NRTI and NNRTI databases, weighted kernels outperformed RF in all cases, whereas their unweighted counterparts did not. This was particularly the case for 3TC, DDI (Fig. 1b), EVG (Fig. 1d) and especially NVP (Fig. 1c), where weighting decreased the Jaccard kernel error by around 50%. In contrast, the effect of weighting was less marked in the PI database: similar errors were obtained (e.g. Figure 1a) for all drugs but TPV, where the error actually increased. In the INI database, weighting decreased dramatically the error in RAL and EVG drugs but not in DTG. In summary, Jaccard was the best weighted kernel followed by Overlap, RBF and Linear.

### Factors affecting prediction error

To investigate the relevance of each factor in prediction, we fitted the following linear model to NMSE obtained in each replicate across all kernels and drugs (40 replicates × 21 drugs × 8 kernels):

9 $$ NMSE\sim N+K+W+ GINI+\varepsilon $$

where *N* is the drug data size (Table 1), *K* is a class variable with the kernel used (Linear, RBF, Overlap or Jaccard), *W =* 0 or 1 depending on whether the kernel was unweighted or weighted, respectively, and *GINI* is the standardized Gini index of RF weights. Table 2 summarizes the coefficients and their significance. We found that all factors are significant and behave additively (interactions were not significant; results not shown). As expected NMSE decreases with *N* but, interestingly, also with Gini index, i.e., prediction improves when there are only a few positions of large effect. Categorical kernels were consistently better than noncategorical ones and Jaccard was the best option in all cases. Weighting protein positions significantly lowers the error, although only in reverse transcriptase and integrase inhibitors (as also observed in Fig. 1 and Additional file 1: Figures S1-S17).

**Table 2:** Linear model coefficient estimates and *p*-values

	All drugs	PIs	NRTIs + NNRTIs	INIs
N increment	−2.1·10^− 4^ ***	−2.0·10^− 5^ ***	−1.8·10^− 4^ ***	−1.3·10^− 3^ ***
Unweighted → Weighted	−2.0·10^− 2^ ***	2.0·10^− 3^	−3.4·10^− 2^ ***	−3.1·10^− 2^ ***
Gini Index increment	−4.9·10^− 3^ ***	−6.0·10^− 2^ ***	−5.4·10^− 2^ ***	1.7·10^−2^ **
Jaccard → Linear	4.5·10^−2^ ***	2.1·10^−2^ ***	5.0·10^− 2^ ***	9.4·10^− 2^ ***
Jaccard → RBF	2.5·10^− 2^ ***	1.3·10^− 2^ ***	2.8·10^− 2^ ***	4.7·10^− 2^ ***
Jaccard → Overlap	1.9·10^− 2^ ***	9.3·10^− 3^ ***	2.1·10^− 2^ ***	3.6·10^− 2^ ***

Legend: Significance codes are 0 *** 0.001 ** 0.01 * 0.05

**Fig. 4:**
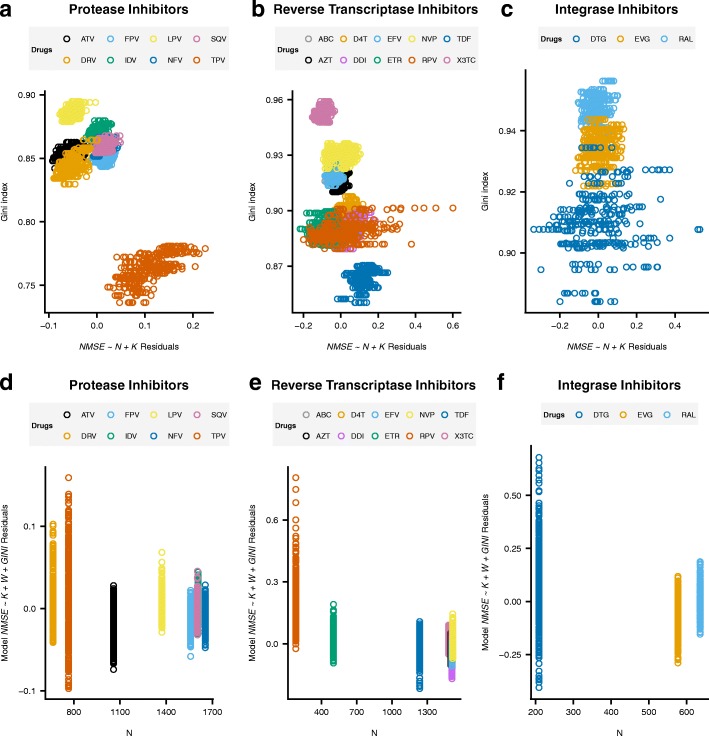
**a, b and c** NMSE residuals (*observed − fitted* values) of the linear model containing only data size (*N*) and kernel (*K*) vs. Gini index. Each color represents a different drug. Note different scale for the Gini index between panels. **d, e and f** Residuals (*observed − fitted* values) of the linear model containing *K, W* and *GINI* vs. data size (*N*). Each color represents a different drug

To visualize the impact of Gini index not ascribable to the effects of data size (*N*) and the kernel used (*K)*, we plotted the residuals of model NMSE ~ *N + K + ε* against *GINI* (Fig. 4 panels a, b and c). For protease inhibitors, the Gini effect is confined to TPV drug (red dots in Fig. 4a). The effect is rather linear for reverse transcriptase inhibitors, although NMSE variability was larger than average for RPV (red dots), the drug with lowest N. In the case of integrase inhibitors, Gini takes values in a narrow range and does not seem to have an impact on prediction. As in the case of RPV, large variability in NMSE values is observed in DTG (blue dots), which is the drug with second lowest sample size.

Sample size is one of the most important factors in any experimental design, and the main one influencing total cost. Figure 4 panels d, e and f show the residuals of model *NMSE* ~ *K + W + GINI* vs. *N*. Although Table 2 shows that the NMSE decreases with sample size for all drugs and proteins, a clear trend appears only for reverse transcriptase inhibitors. In this case, a law of diminishing returns is observed, and adjusted NMSE decrease with N is very small for N > ~ 600.

### Kernel PCA

**Fig. 5:**
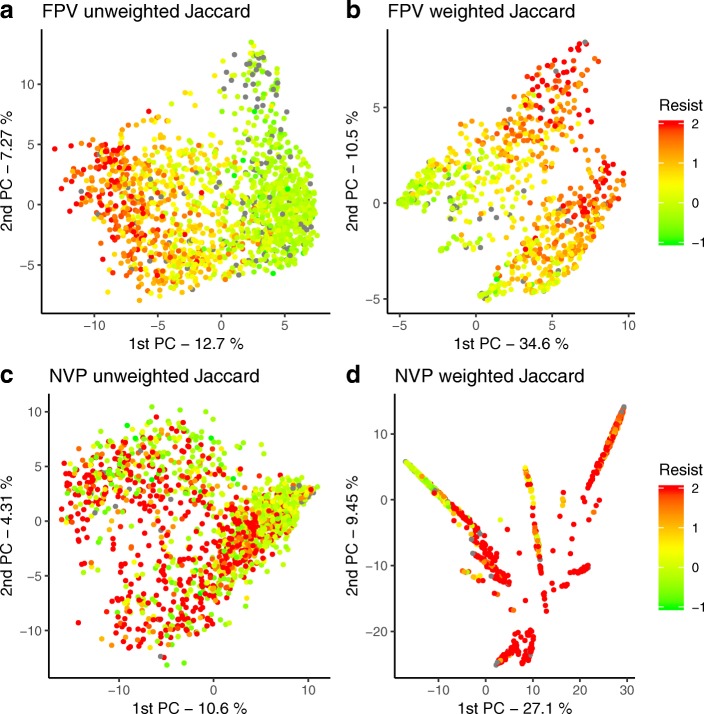
The Jaccard kernel PCA in a protease inhibitor (FPV, panels **a** and **b**) and a reverse transcriptase inhibitor (NVP, panels **c** and **d**). Panels **a** and **c** correspond to unweighted Jaccard, and **b** and **d** to weighted Jaccard. Dot color represents the actual log-resistance value for each specific drug; in red the more resistant, and in green the least resistant. Sequences with missing resistance value are in gray

Even if weighting increases prediction accuracy overall, the effect was markedly different when we compare reverse transcriptase and integrase with protease (Table 2). In the latter protein, weighted kernels were not clearly superior. To further investigate this issue, we performed a PCA on the Jaccard kernel. Figure 5 shows the results of for FPV (a protease inhibitor, panels a and b) and NVP (a reverse transcriptase inhibitor, panel c and d), both with unweighted and weighted Jaccard kernels. The remaining figures can be found at (Additional file 4: Figures S36-S54). Unweighted kernel PCA results, overall, in a good, spectrum-like separation between resistant and susceptible isolates for protease inhibitors, whereas weighted kernels can improve dramatically the separation in the case of reverse transcriptase. The integrase inhibitors RAL and EVG behave similarly to reverse transcriptase inhibitors, while DTG (which has a very small sample size) do not achieve a good separation either in the weighted or the unweighted kernel PCAs.

### Stacked models

We compared the performances of four methods (SVM plus weighted Linear, RBF, Overlap and Jaccard kernels) with those of their stacked counterparts in Additional file 3: Tables S1 (mean NMSE) and S2 (NMSE standard error). Intriguingly, we found that the stacked versions of SVM with weighted kernels have similar performances to those of the individual models. This suggests that all the information of the sequence has been already extracted in the first step, and so stacking the models was of no additional value.

### Performance comparison to other approaches

**Fig. 6:**
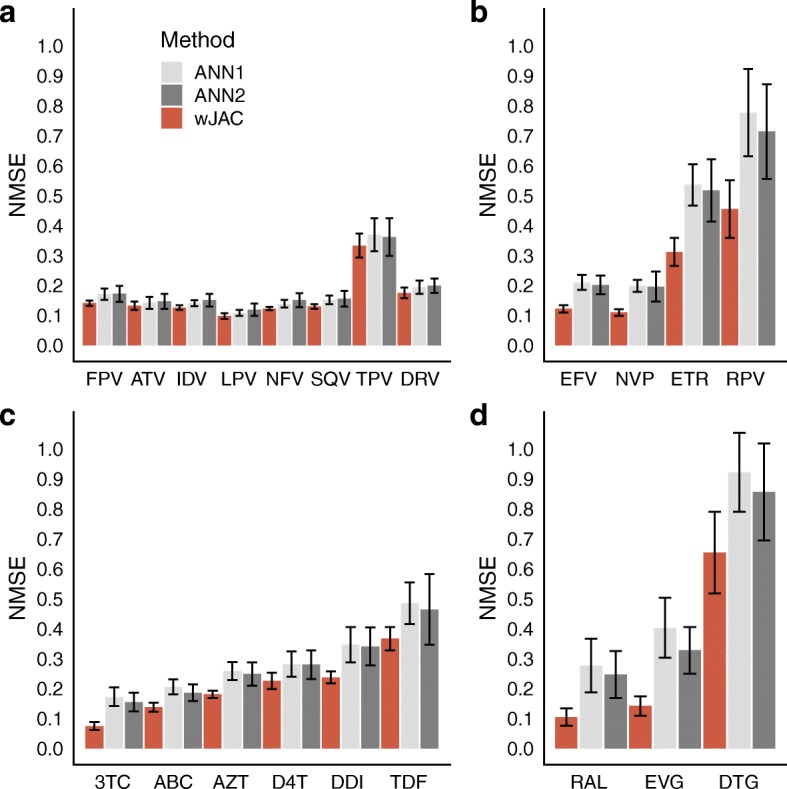
Mean NMSE values and their corresponding standard errors for the SVM + weighted Jaccard kernels (red), ANN1 (light gray) and ANN2 (dark gray). PIs are shown in panel **a**, NRTIs in panel **c**, NNRTIs in panel **b** and INIs in panel **d**

Figure 6 shows the performance comparison between our best method (SVM with weighted Jaccard kernel) with the ANN1 and ANN2 (see “Performance comparison to other approaches” in Material and methods). ANN2 tends to have better performance than ANN1, especially in drugs with small sample size, but also presents greater standard errors in some drugs. In the case of protease inhibitors (panel a) both ANN1 and ANN2 are only marginally worse than the weighted Jaccard SVM, with the exception of the FPV drug. In the case of reverse transcriptase and the integrase inhibitors (panels b, c and d), the difference between the performance of weighted Jaccard and the ANN increases. The latter method presents higher NMSE and larger standard errors, especially for 3TC, DDI, TDF, the NNRTIs, and the INIs.

## Discussion

Recent results on predicting HIV drug resistance as a regression problem can be found in [14, 18]. Shen et al. [18] used RF and computed the 5-fold cross-validation R^2^. Sheik Amamuddy et al. [14] used ANN and computed the R^2^ of the test set without replicates. The two approaches were based in a previous version of the Stanford dataset (version date: 2014-9-28) and share a similar treatment of amino acid mixtures based on sequence expansions. We did a comparison with the ANN, which to our knowledge achieved the best performance so far in this dataset [14]. We observed that weighted Jaccard outperforms ANN in all drugs, and that the ANN prediction performances were worse than those originally reported (which had R^2^ values ranging between of 0.85 and 0.99). It has to be stressed, however, that we used different versions of the dataset (the version used by [14], for instance, did not contain information about the INIs) and that we followed very different strategies concerning pre-processing. In [14] a pre-processing with removal of outliers and rare variant filtering is performed, which can result in a loss of generalizability, as is acknowledged by the authors. Another reason for the discrepancy is probably the treatment of allele mixtures, as we discuss next.

In this work, we present a novel approach to predict drug resistance in HIV, using kernel functions that directly address the presence of allele mixtures and the categorical nature of the data. Previous work handled these two issues using several pre-processing strategies. Categorical data are systematically recoded into numeric data, usually in the form of dummy data or, in [14], assigning an integer to each category. Here, we have shown that addressing the categorical nature of the data and the presence of allele mixtures does lower the test error in comparison to the dummy variable approach (Table 2). In fact, even the simplest categorical kernel (i.e. the Overlap kernel) improves prediction upon the standard RBF kernel, although the extent of the improvement depends on the specific drug. It has to be stressed that recoding the categorical data into dummy variables increases the dimensionality of the problem, thus increasing computation needs and leading to sparse datasets. As this effect depends on the number of different categories of the variables, categorical methods may be more useful when data has more than few categories. Coding the different alleles as an integer does not increase the dimensionality either, but introduces an order without biological meaning among the amino acids.

The treatment of amino acid mixtures is more challenging. In the data analyzed we observed that it is a widespread phenomenon: about 60% of the sequences had at least one mixture. Mixtures introduce ambiguity in the genotype-phenotype correlation since it makes impossible to know the actual sequences of strains. Also, the quasispecies distribution may have undergone undefined modifications during the in vitro assay [28]. Previous approaches to deal with this issue included keeping the most frequent amino acid of the mixture [19] and sequence expansion [11, 14, 18]. The latter strategy consists on expanding the data to sequences with single amino acids at each mixture location until all possible combinations have been exhausted. These “derived” sequences share the resistance value, i.e., the resistance of the original sequence. This approach dramatically enlarges data size (in the aforementioned works, minimum by a 10x factor in the protease inhibitors and almost a 30x in the reverse transcriptase inhibitors). This might be one of the main reasons for the discrepancy between the ANN performance computed in this work and in [14]. Without expansion, the data size ranges between 200 and 1500, but the number of (dummy) variables is almost 2000 in the PIs, and more than 4000 in the other drugs. The higher number of variables compared to observations might have adversely affected the ANN performance in comparison to the original work and, also, in comparison to SVMs, as the latter are less prone to over-fitting. Furthermore, the expansion potentially biases the dataset by over representing sequences with mixtures (especially those with a larger number of mixtures and/or alleles per mixture) and it can generate HIV variants not found in the patient. Expansion also increases the difficulty of the training/test splitting because all expansions of the same sequence must be placed either in the training set or in the test set; otherwise, the independence of both sets is lost. In our work, we preferred keeping only one amino acid of the mixture, which is allegedly the most conservative pre-processing choice. This differs from e.g. [19], because we keep one amino acid at random, while they pick the most frequent one, which is sound if mixtures are considered a technical artifact. However, in case of HIV, this event mostly reflects the coexistence of actual HIV variants in the body of the patient [2, 4, 6, 28] and the ambiguity lies in the resistance value delivered via the in vitro test. In any case, part of the original information is lost by picking one of the allele of the mixture. This does not happen when using the Jaccard kernel, which naturally handles allele mixtures. We have shown that Jaccard is clearly the best among kernels assessed and that also improves the RF results, in most cases by a large margin. Both Overlap and Jaccard are basic kernel functions, but our kernel definition (7) is general enough to replace them for more sophisticated categorical kernels, perhaps with improved prediction performance.

An additional theoretical proposal was to weigh kernel positions according to its inferred influence on drug resistance. Here we employed RF decrease in impurity as weights but numerous options are equally justified and so additional research on this topic is warranted. Using RF we were able to identify, from protein sequence alone, important positions for the drug resistance that have a structural meaning (Fig. 3). We observed a distinct effect of weighting in protease inhibitors and transcriptase reverse inhibitors that correlates with the distribution of the importances. At least part of this behavior might be due to differences in the mutational pattern between the two enzymes in regards to drug resistance. In the reverse transcriptase, the major resistance mutations tend to be located in specific positions, particularly at the drug binding sites of the N-terminal side, weakening the affinity between drug and enzyme. As early as 1998, it was noted that a single mutation of the reverse transcriptase may confer high resistance to drugs like 3TC and NVP [28], whereas the virus acquires resistance to protease inhibitors by accumulating mutations. First, primary resistance mutations arise at the active site pocket and the surrounding residues. But, as these mutations often cause conformational changes, additional secondary mutations that compensate the impaired catalytic activity and stabilize the protease tend to be selected in turn [2]. There are at least 36 important residues (out of a total of 99) involved in protease drug resistance mutations and (unlike reverse transcriptase) they are distributed along the whole sequence [2]. These differences may explain why RF, and therefore the weighted categorical kernels, performed better at the NRTI and NNRTI databases. Further, the estimate of the variable importance is more reliable when few relevant protein positions have a large impact on resistance. In contrast, the compensatory secondary mutations of the protease probably introduce some degree of correlation between protein positions, which may explain why weighting in PI database does not result in a clear improvement of performance.

## Conclusions

Machine learning is an effective approach to predict HIV drug resistance, and a straightforward alternative to the much slower and expensive in vitro assay. Results show that kernels that take into account both the categorical nature of the data and the presence of mixtures consistently result in the best prediction model. As for the introduction of position weights, we found that the amount of improvement was a function of the number of positions with large effect on drug resistance, which may be related to the known different mutational patterns regarding drug resistance among the viral proteins. Using more sophisticated categorical kernels and/or kernels able to take into account structural information may improve even more the resistance prediction.

## Additional files

Additional file 1: **Figures S1-S17**. NMSE distribution for drugs ATV, DRV, IDV, LPV, NFV, TPV, SQV, 3TC, ABC, AZT, D4T, TDF, EFV, ETR, RPV, RAL and DTG (PDF 671 kb)

Additional file 2: **Figures S18-S35**. RF weights for drugs ATV, DRV, IDV, LPV, NFV, TPV, SQV, 3TC, ABC, AZT, D4T, DDI, TDF, EFV, ETR, RPV, DTG and RAL (PDF 530 kb)

Additional file 3: **Table S1**. Average NMSE of stacked methods**. Table S2.** NMSE Standard error of stacked methods (PDF 42 kb)

Additional file 4: **Figures S36-S54**. Kernel PCAs for drugs ATV, DRV, IDV, LPV, NFV, TPV, SQV, 3TC, ABC, AZT, D4T, DDI, TDF, EFV, ETR, RPV, DTG, EVG and RAL (PDF 2075 kb)

## Data Availability

The datasets analyzed during the current study are available in the Genotype-Phenotype Stanford HIV Drug Resistance Database repository, https://hivdb.stanford.edu/pages/genopheno.dataset.html. Structural data can be found at https://www.rcsb.org/structure/3ekx and https://www.rcsb.org/structure/3v81. Code used in this manuscript is available at https://bitbucket.org/elies_ramon/catkern.

## Abbreviations

3TC: Lamivudine
ABC: Abacavir
AIDS: Acquired immunodeficiency syndrome
ANN: Artificial Neural Networks
ATV: Atazanavir
AZT: Zidovudine
BIC: Bictegravir
CAB: Cabotegravir
D4T: Stavudine
DDI: Didanosine
DRV: Darunavir
DT: Decision Trees
DTG: Dolutegravir
EFV: Efavirenz
ETR: Etravirine
EVG: Elvitegravir
FPV: Fosamprenavir
HIV: Human immunodeficiency virus
IC50: Half maximal inhibitory concentration
IDV: Indinavir
INI: Integrase inhibitor
LPV: Lopinavir
NFV: Nelfinavir
NMSE: Normalized Mean Square Error
NNRTI: Non-nucleoside reverse transcriptase inhibitors
NRTI: Nucleoside reverse transcriptase inhibitors
NVP: Nevirapine
PCA: Principal Components Analysis
PI: Protease inhibitors
RAL: Raltegravir
RF: Random Forests
RPV: Rilpivirine
SQV: Saquinavir
SVM: Support Vector Machine
TDF: Tenofovir
TPV: Tipranavir
WHO: World Health Organization
