# Supplementary material for: HIV drug resistance prediction with weighted categorical kernel functions
Source: BMC Bioinformatics. 2019 Jul 30;20:410. doi: 10.1186/s12859-019-2991-2 (PMC6668108; doi:10.1186/s12859-019-2991-2)
Supplement: Supplementary file 1 — Figures S1-S17. NMSE distribution for drugs ATV, DRV, IDV, LPV, NFV, TPV, SQV, 3TC, ABC, AZT, D4T, TDF, EFV, ETR, RPV, RAL and DTG (PDF 671 kb) [file 12859_2019_2991_MOESM1_ESM.pdf]

ADDITIONAL FILE 1

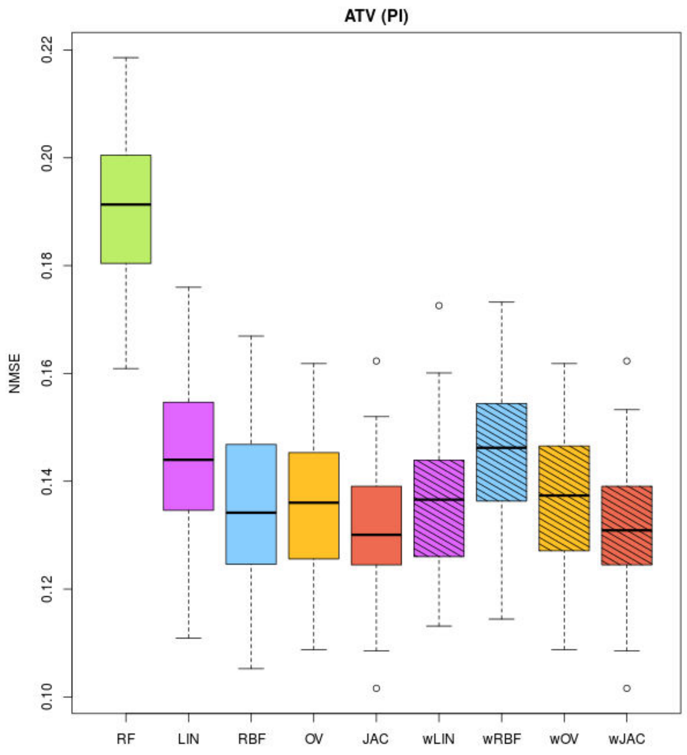

Fig. S1. NMSE distribution for ATV (protease inhibitor). Same legend as that of Fig. 1.

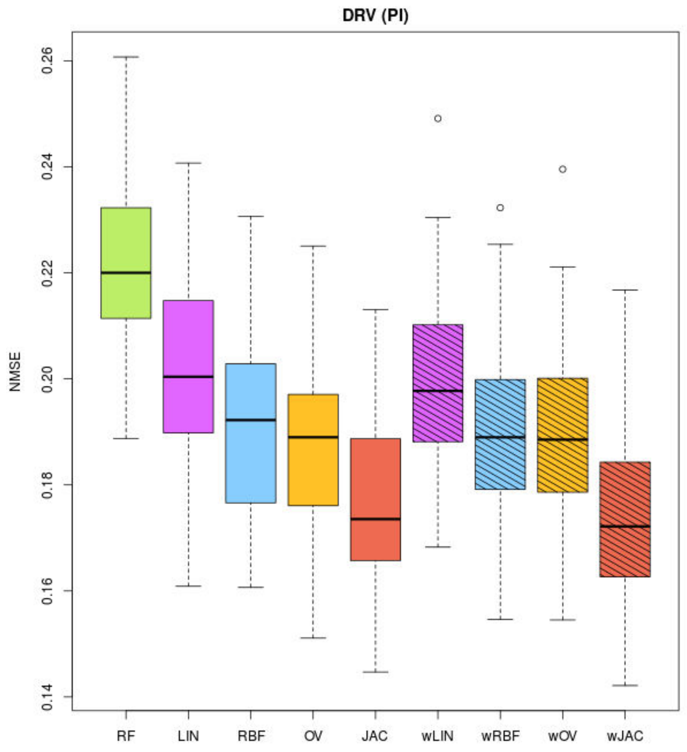

Fig. S2. NMSE distribution for DRV (protease inhibitor). Same legend as that of Fig. 1.

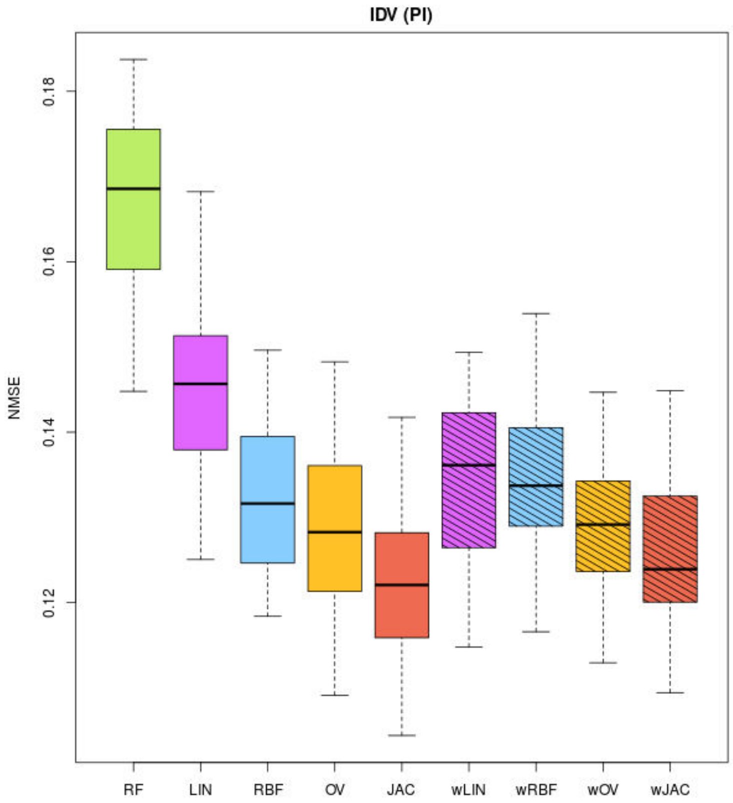

Fig. S3. NMSE distribution for IDV (protease inhibitor). Same legend as that of Fig. 1.

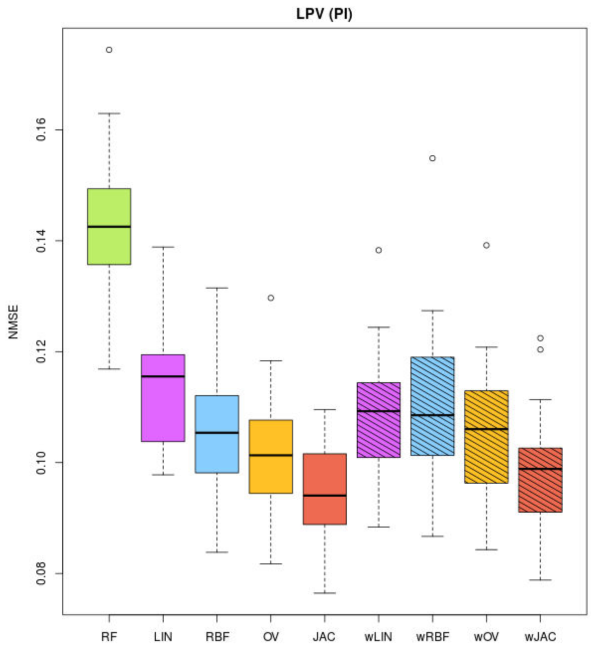

Fig. S4. NMSE distribution for LPV (protease inhibitor). Same legend as that of Fig. 1.

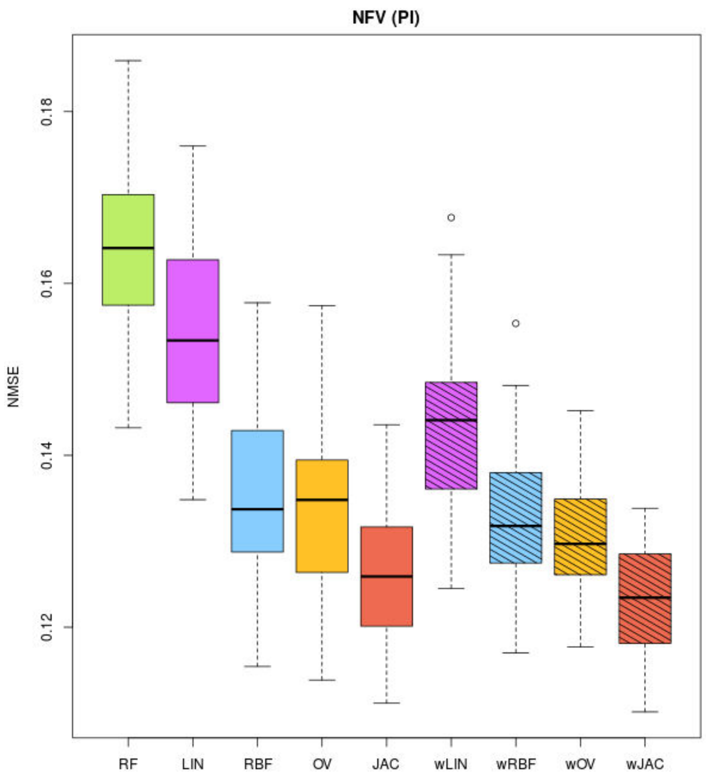

Fig. S5. NMSE distribution for NFV (protease inhibitor). Same legend as that of Fig. 1.

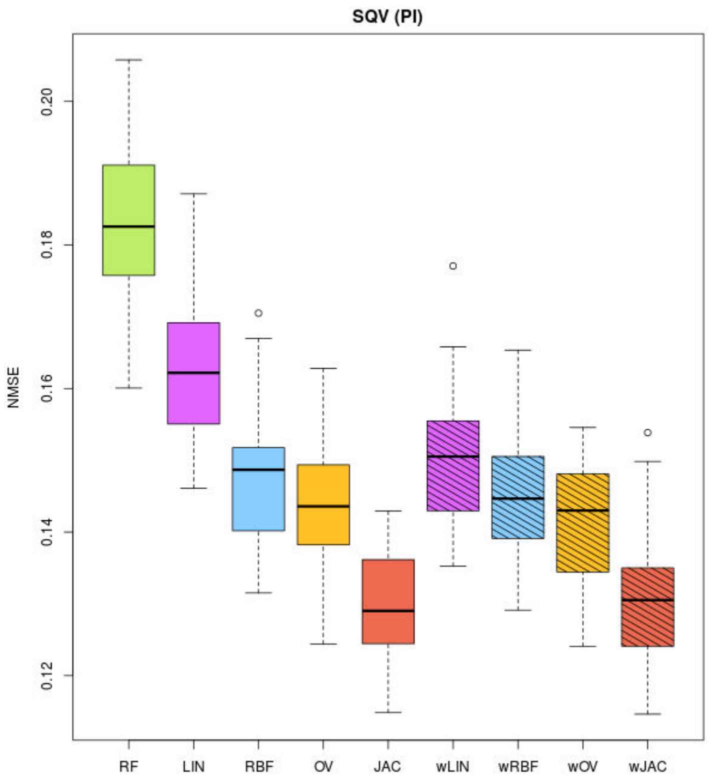

Fig. S6. NMSE distribution for SQV (protease inhibitor). Same legend as that of Fig. 1.

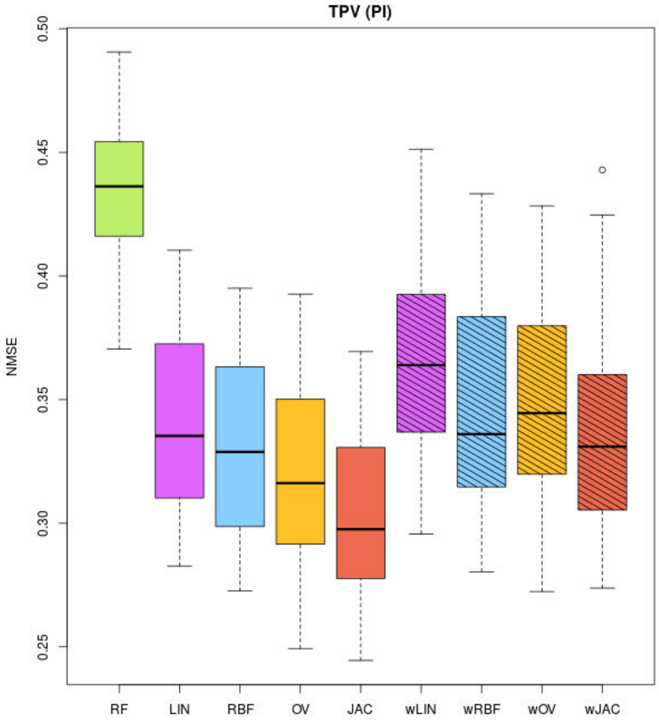

Fig. S7. NMSE distribution for TPV (protease inhibitor). Same legend as that of Fig. 1.

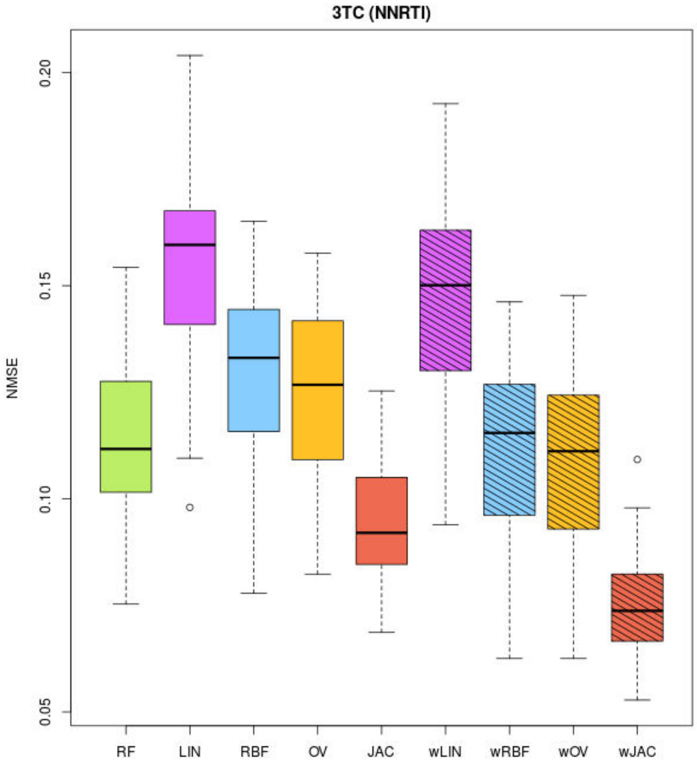

Fig. S8. NMSE distribution for 3TC (reverse transcriptase inhibitor). Same legend as that of Fig. 1.

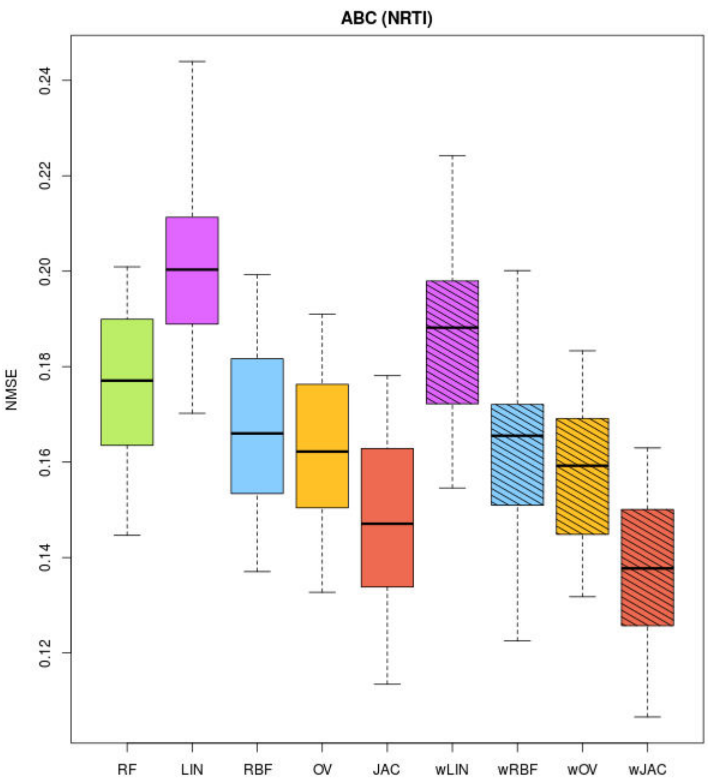

Fig. S9. NMSE distribution for ABC (reverse transcriptase inhibitor). Same legend as that of Fig. 1.

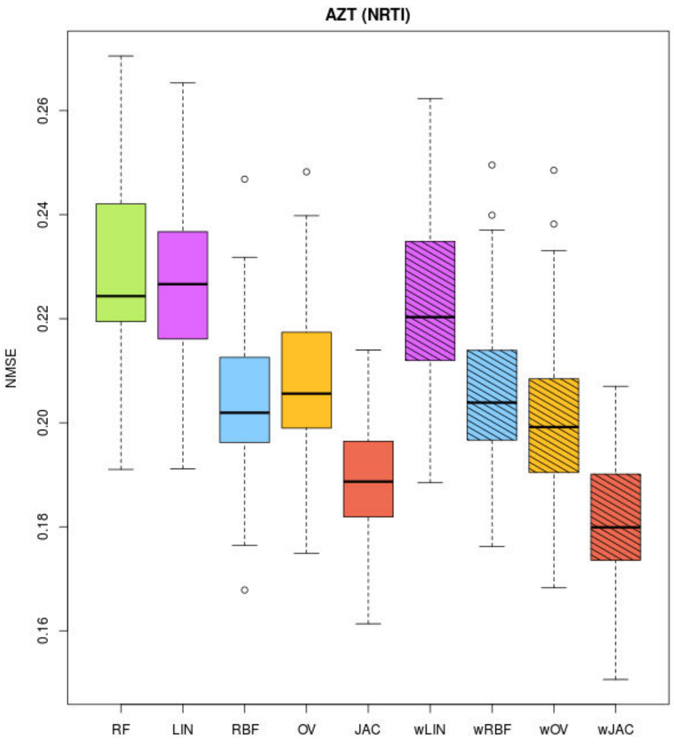

Fig. 10. NMSE distribution for AZT (reverse transcriptase inhibitor). Same legend as that of Fig. 1.

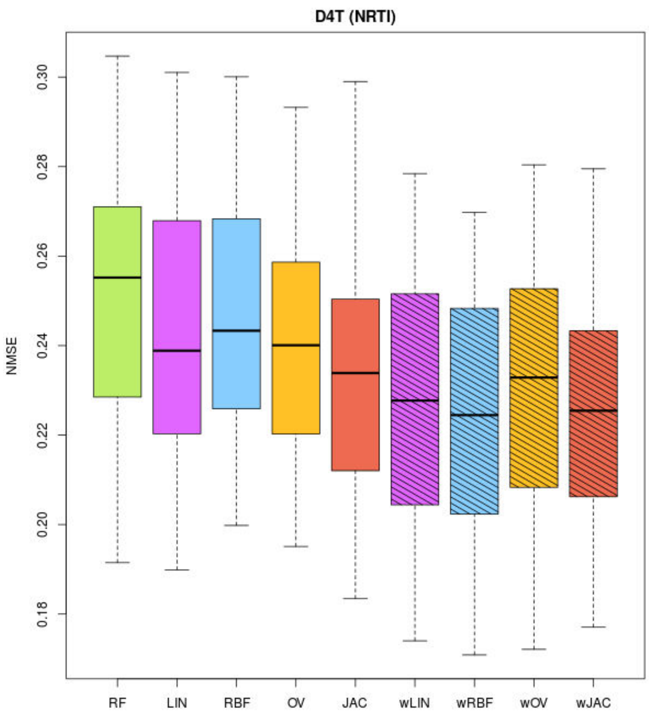

Fig. S11. NMSE distribution for D4T (reverse transcriptase inhibitor). Same legend as that of Fig. 1.

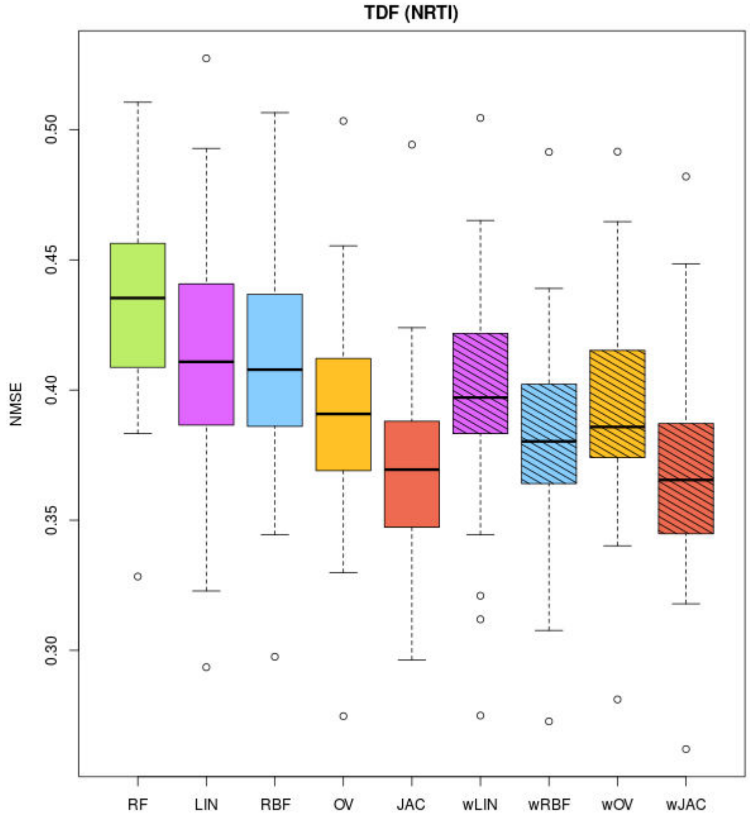

Fig. S12. NMSE distribution for TDF (reverse transcriptase inhibitor). Same legend as that of Fig. 1.

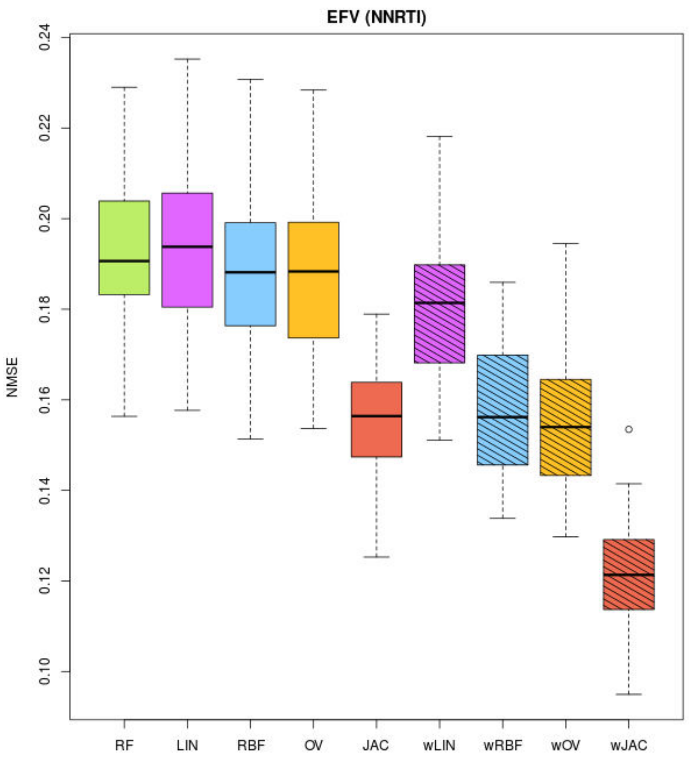

Fig. S13. NMSE distribution for EFV (reverse transcriptase inhibitor). Same legend as that of Fig. 1.

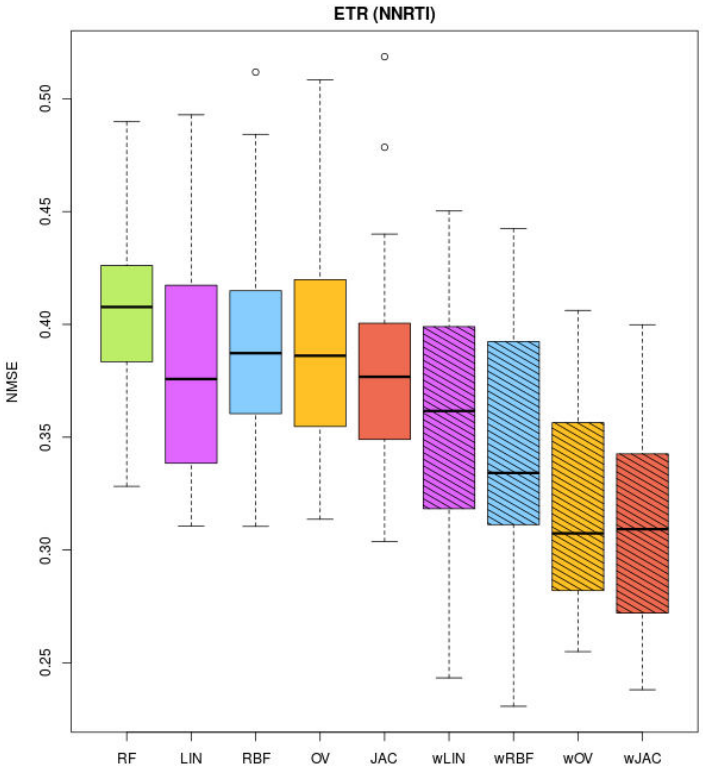

Fig. S14. NMSE distribution for ETR (reverse transcriptase inhibitor). Same legend as that of Fig. 1.

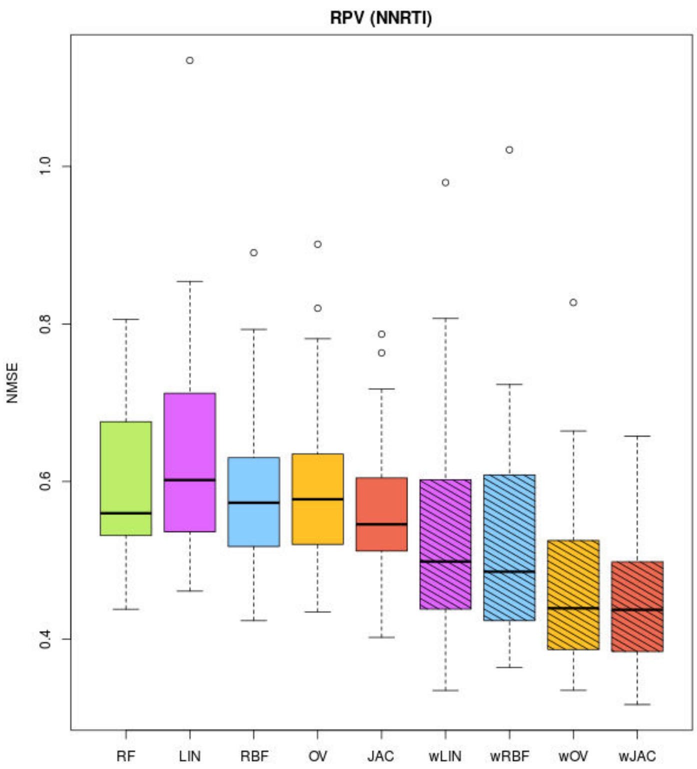

Fig. S15. NMSE distribution for RPV (reverse transcriptase inhibitor). Same legend as that of Fig. 1.

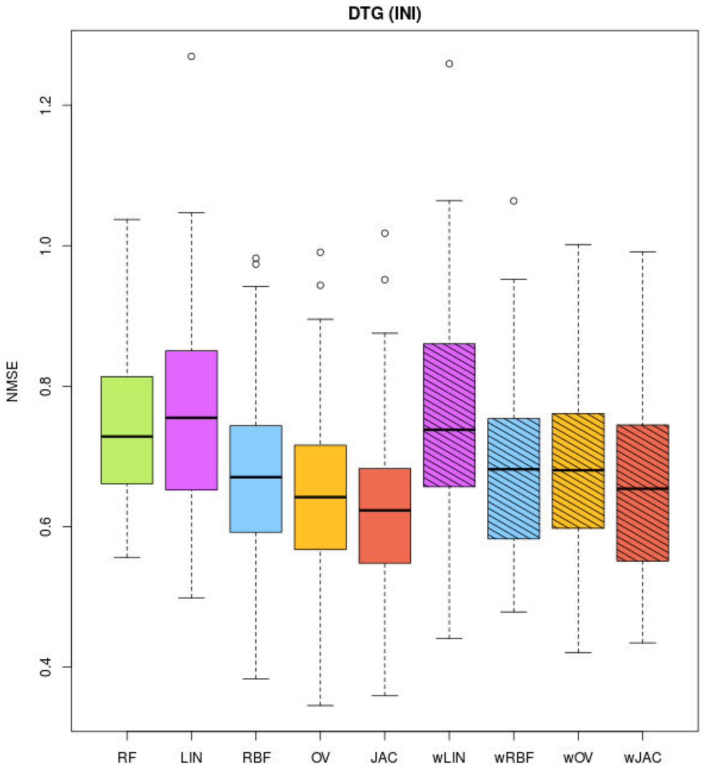

Fig. S16. NMSE distribution for DTG (integrase inhibitor). Same legend as that of Fig. 1.

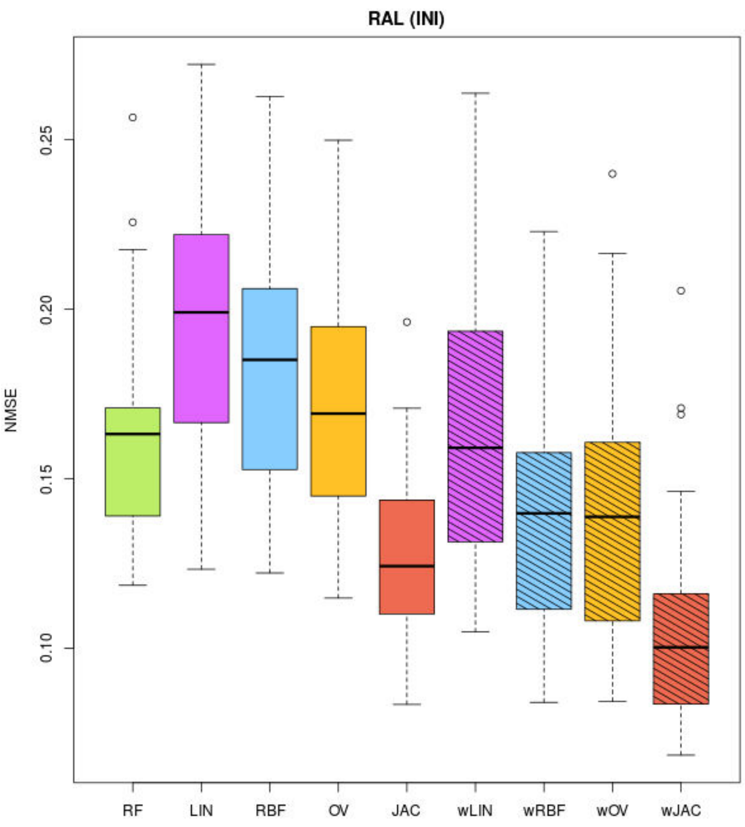

Fig. S17. NMSE distribution for RAL (integrase inhibitor). Same legend as that of Fig. 1.
