# Supplementary material for: HIV drug resistance prediction with weighted categorical kernel functions
Source: BMC Bioinformatics. 2019 Jul 30;20:410. doi: 10.1186/s12859-019-2991-2 (PMC6668108; doi:10.1186/s12859-019-2991-2)
Supplement: Supplementary file 2 — Figures S18-S35. RF weights for drugs ATV, DRV, IDV, LPV, NFV, TPV, SQV, 3TC, ABC, AZT, D4T, DDI, TDF, EFV, ETR, RPV, DTG and RAL (PDF 530 kb) [file 12859_2019_2991_MOESM2_ESM.pdf]

ADDITIONAL FILE 2

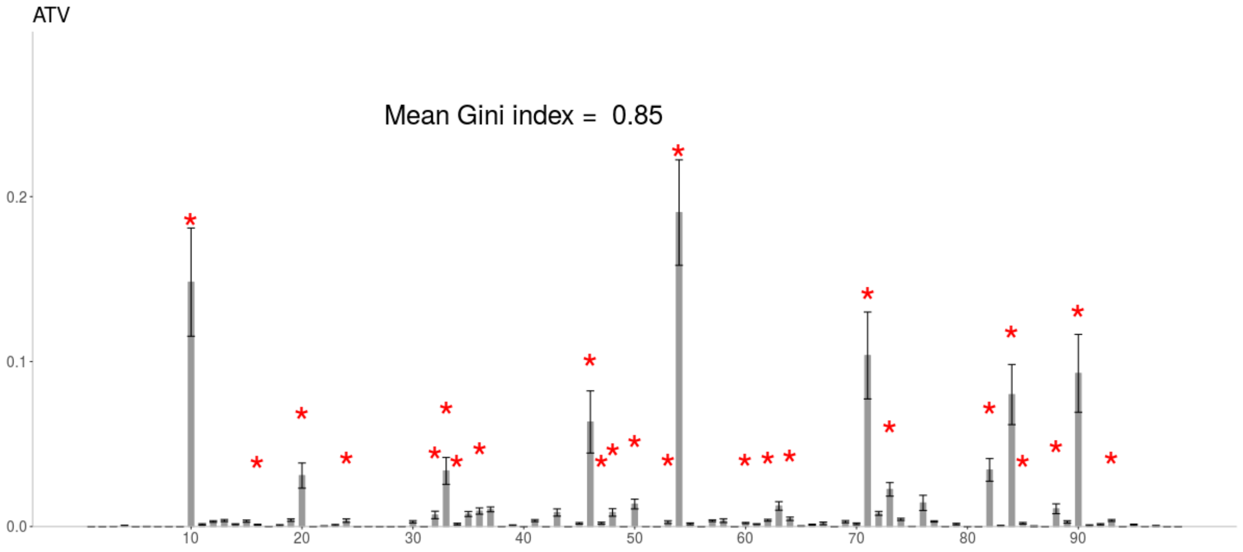

Fig. S18. RF relative importance of each protein position, averaged over 40 replicates, for ATV (protease inhibitor). Asterisks mark the major drug-related positions reported in the literature.

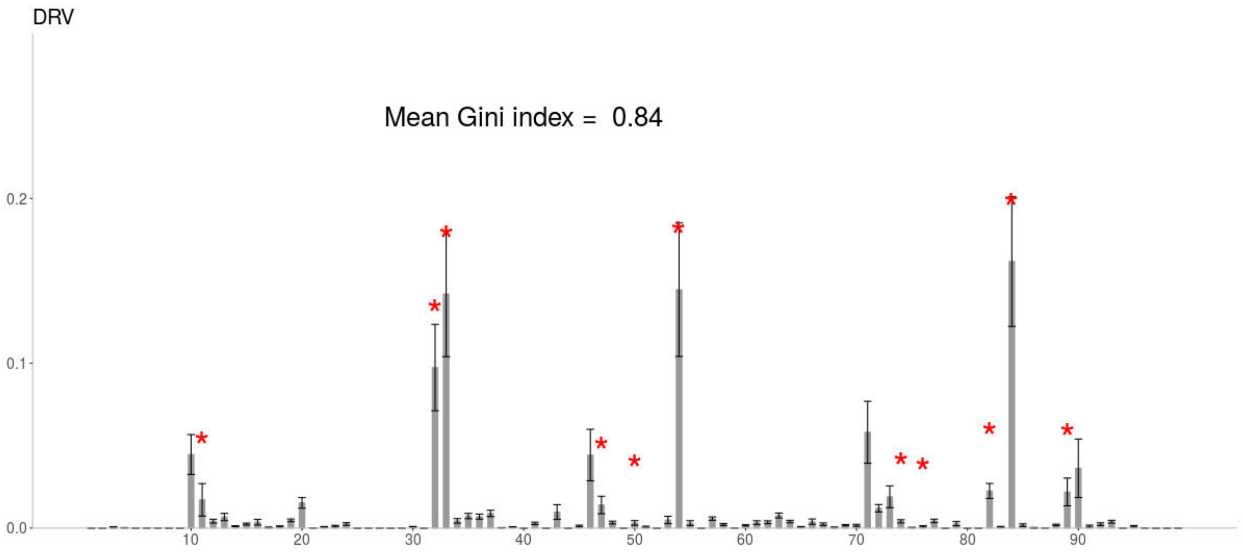

Fig. S19. RF relative importance of each protein position, averaged over 40 replicates, for DRV (protease inhibitor). Asterisks mark the major drug-related positions reported in the literature.

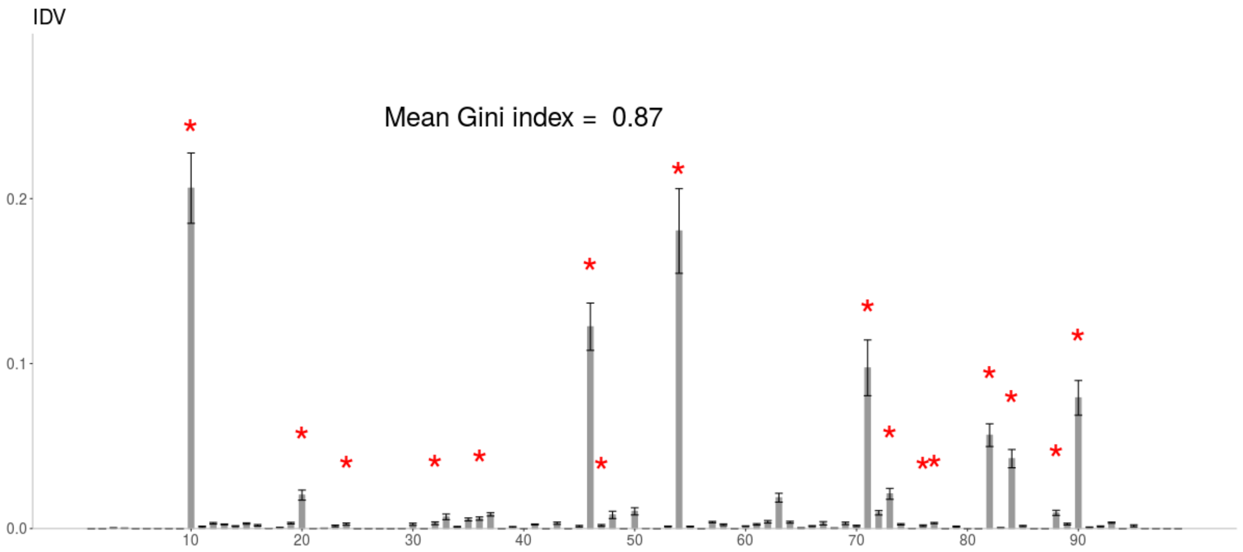

Fig. S20. RF relative importance of each protein position, averaged over 40 replicates, for IDV (protease inhibitor). Asterisks mark the major drug-related positions reported in the literature.

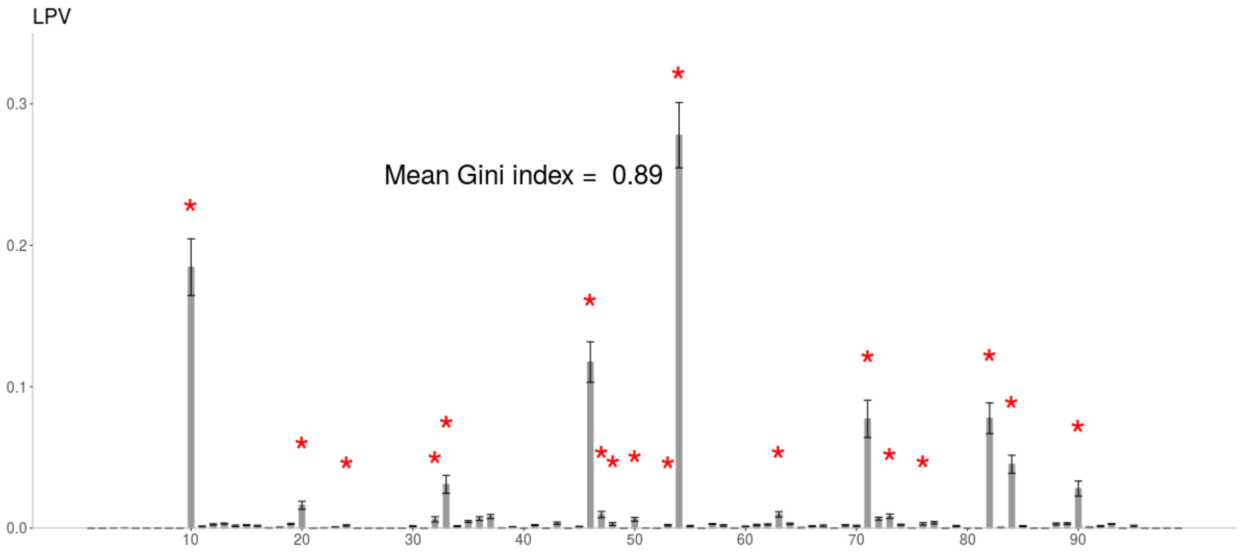

Fig. S21. RF relative importance of each protein position, averaged over 40 replicates, for LPV (protease inhibitor). Asterisks mark the major drug-related positions reported in the literature.

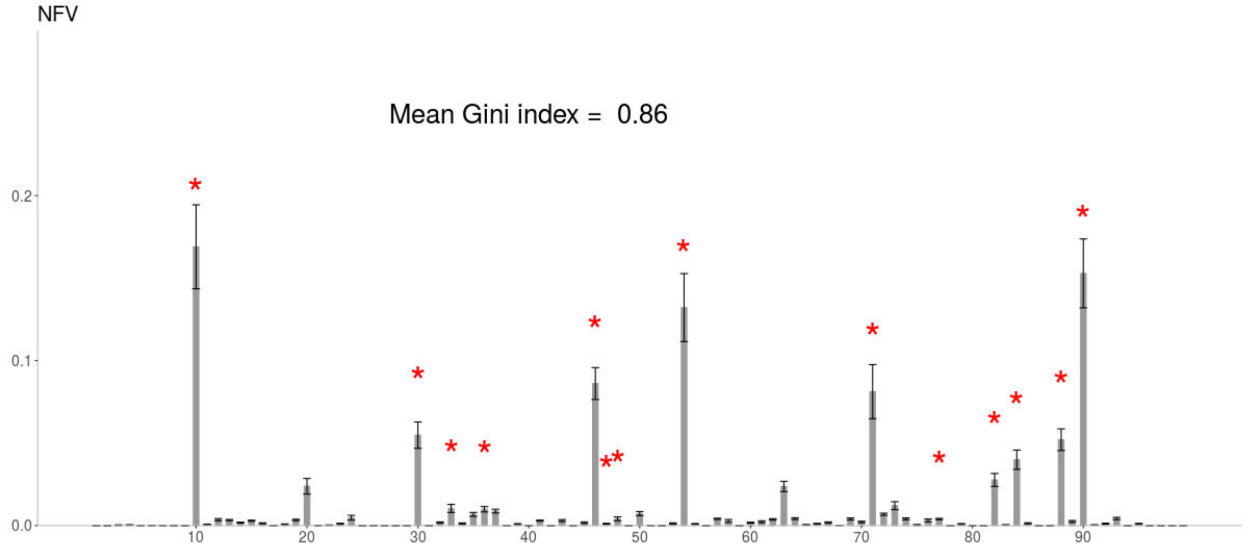

Fig. S22. RF relative importance of each protein position, averaged over 40 replicates, for NFV (protease inhibitor). Asterisks mark the major drug-related positions reported in the literature.

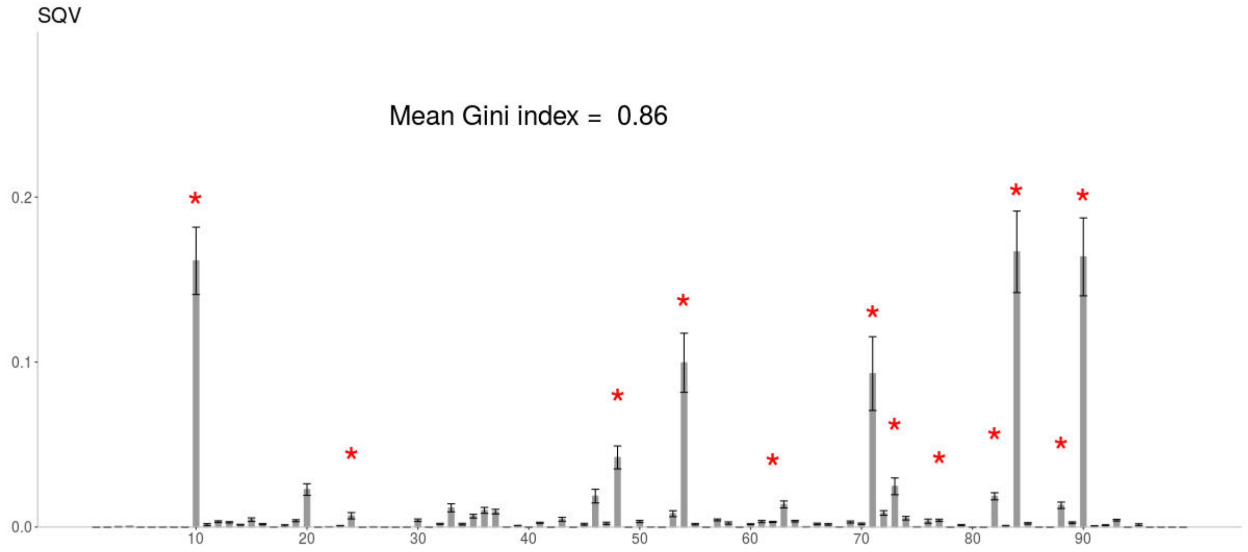

Fig. S23. RF relative importance of each protein position, averaged over 40 replicates, for SQV (protease inhibitor). Asterisks mark the major drug-related positions reported in the literature.

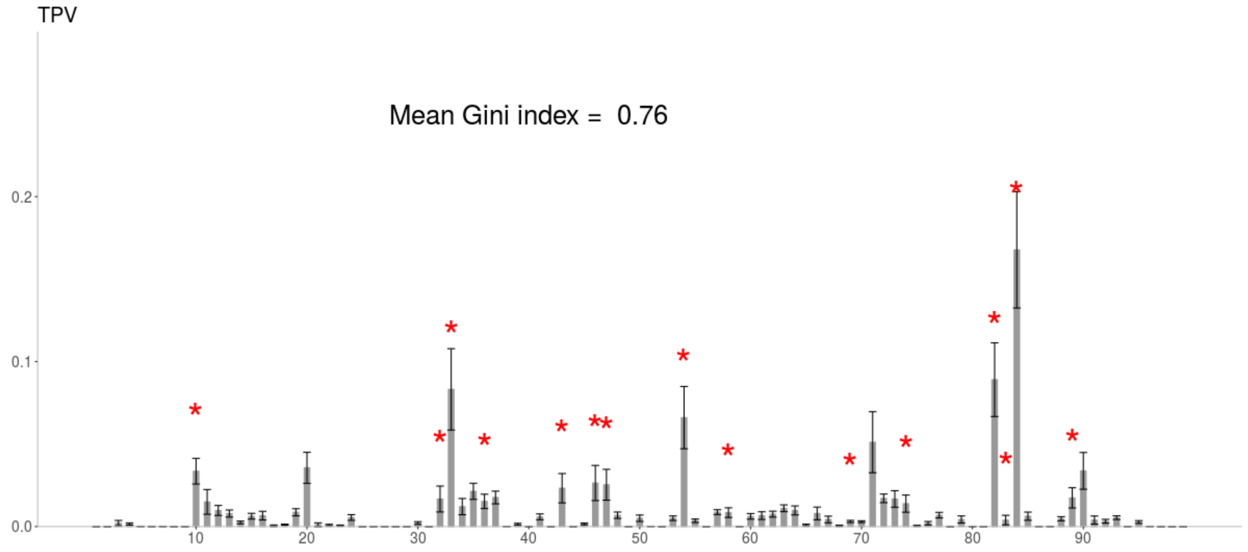

Fig. S24. RF relative importance of each protein position, averaged over 40 replicates, for TPV (protease inhibitor). Asterisks mark the major drug-related positions reported in the literature.

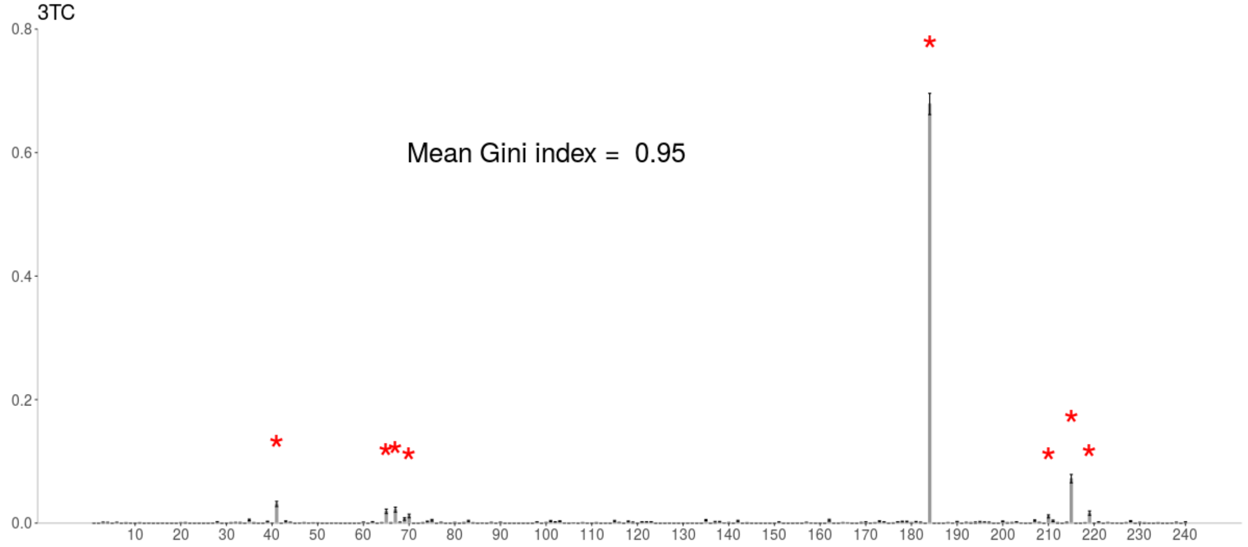

Fig. S25. RF relative importance of each protein position, averaged over 40 replicates, for 3TC (reverse transcriptase inhibitor). Asterisks mark the major drug-related positions reported in the literature.

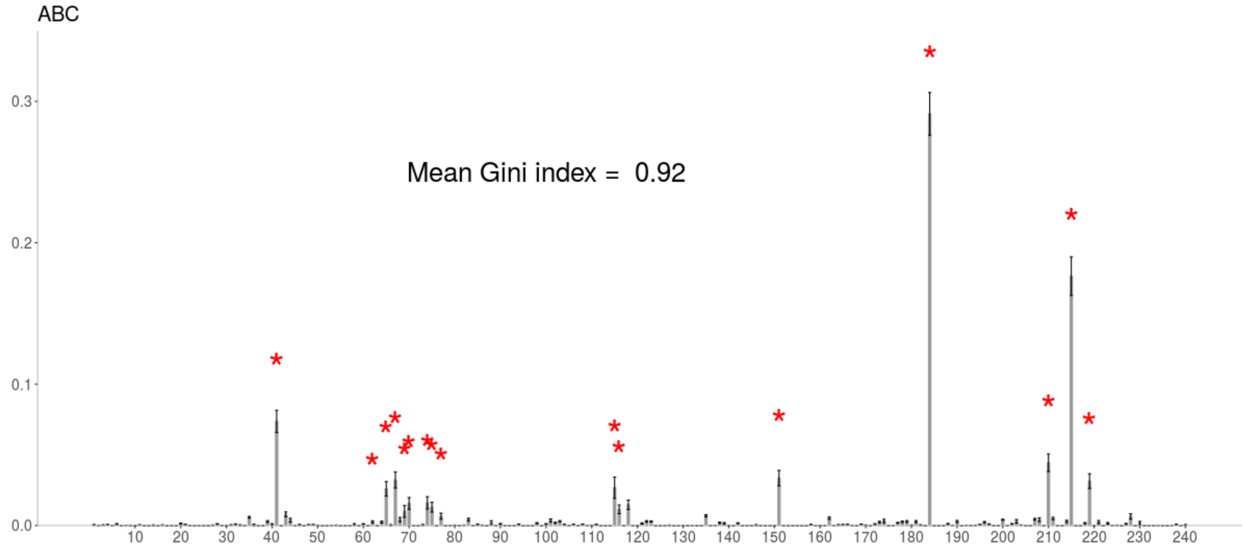

Fig. S26. RF relative importance of each protein position, averaged over 40 replicates, for ABC (reverse transcriptase inhibitor). Asterisks mark the major drug-related positions reported in the literature.

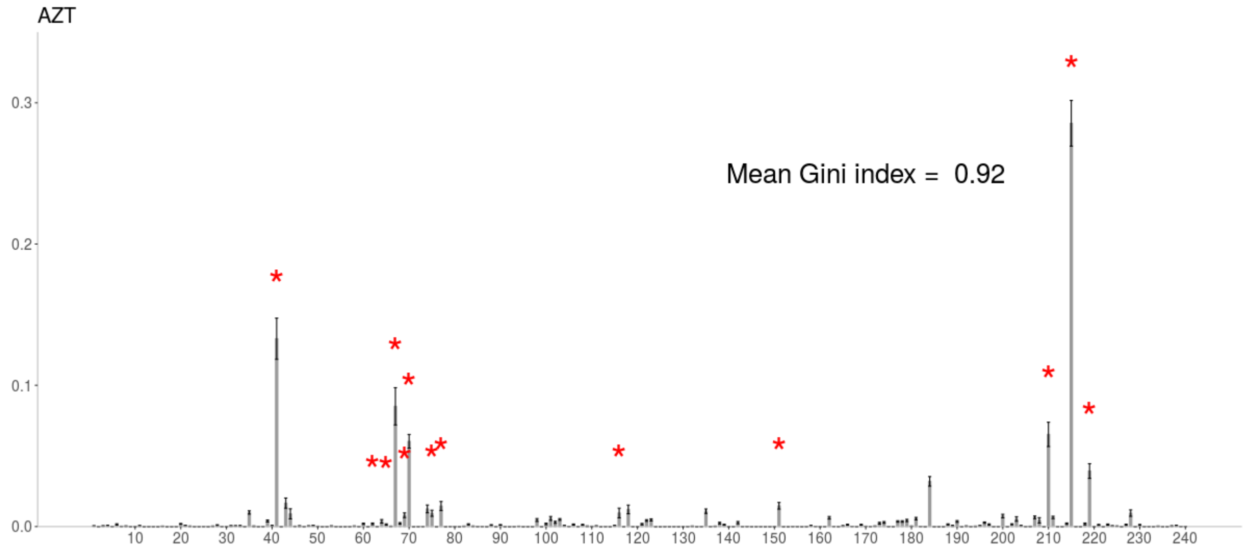

Fig. S27. RF relative importance of each protein position, averaged over 40 replicates, for AZT (reverse transcriptase inhibitor). Asterisks mark the major drug-related positions reported in the literature.

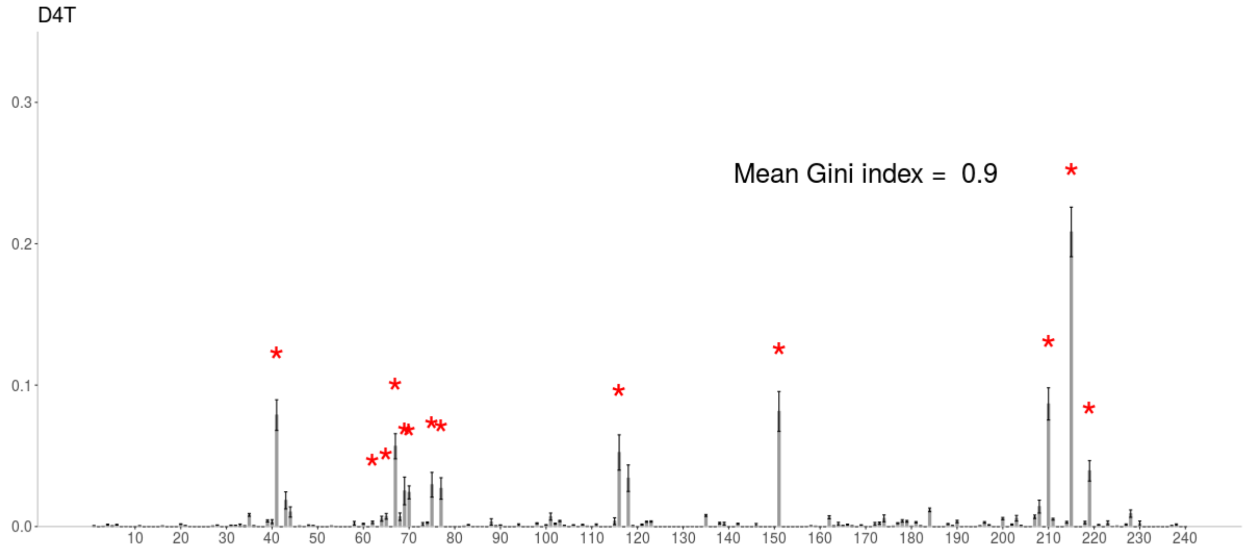

Fig. S28. RF relative importance of each protein position, averaged over 40 replicates, D4T (reverse transcriptase inhibitor). Asterisks mark the major drug-related positions reported in the literature.

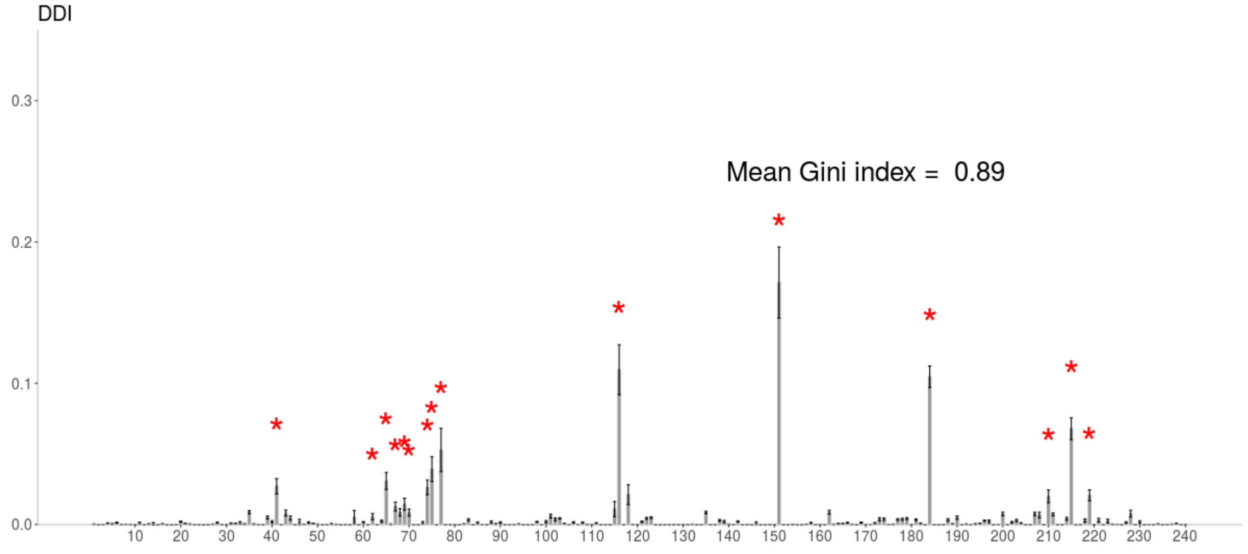

Fig. S29. RF relative importance of each protein position, averaged over 40 replicates, for DDI (reverse transcriptase inhibitor). Asterisks mark the major drug-related positions reported in the literature.

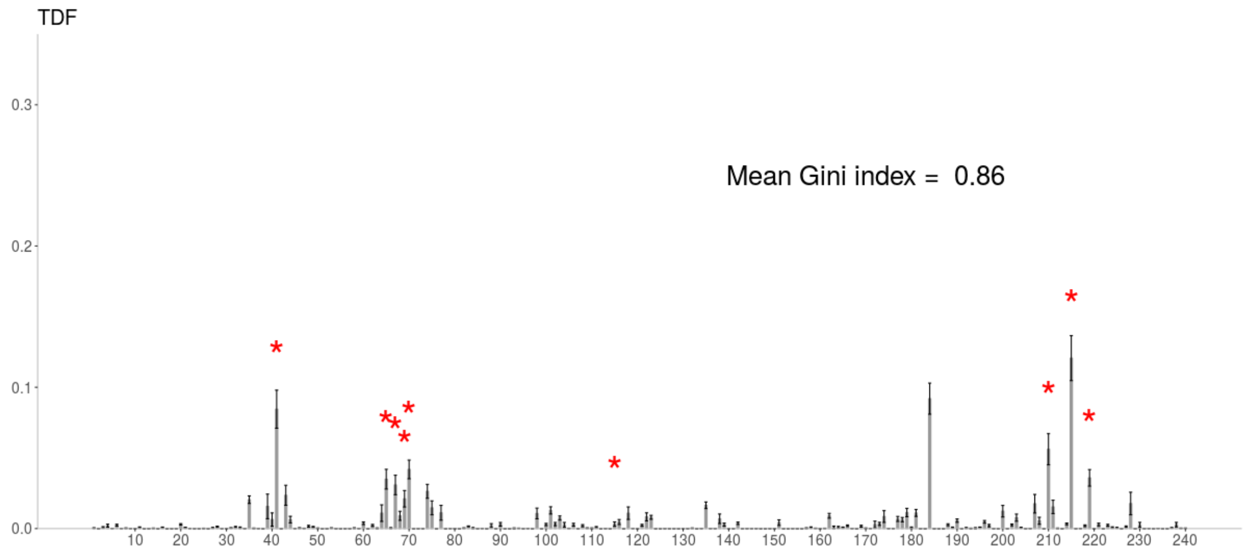

Fig. 30. RF relative importance of each protein position, averaged over 40 replicates, for TDF (reverse transcriptase inhibitor). Asterisks mark the major drug-related positions reported in the literature.

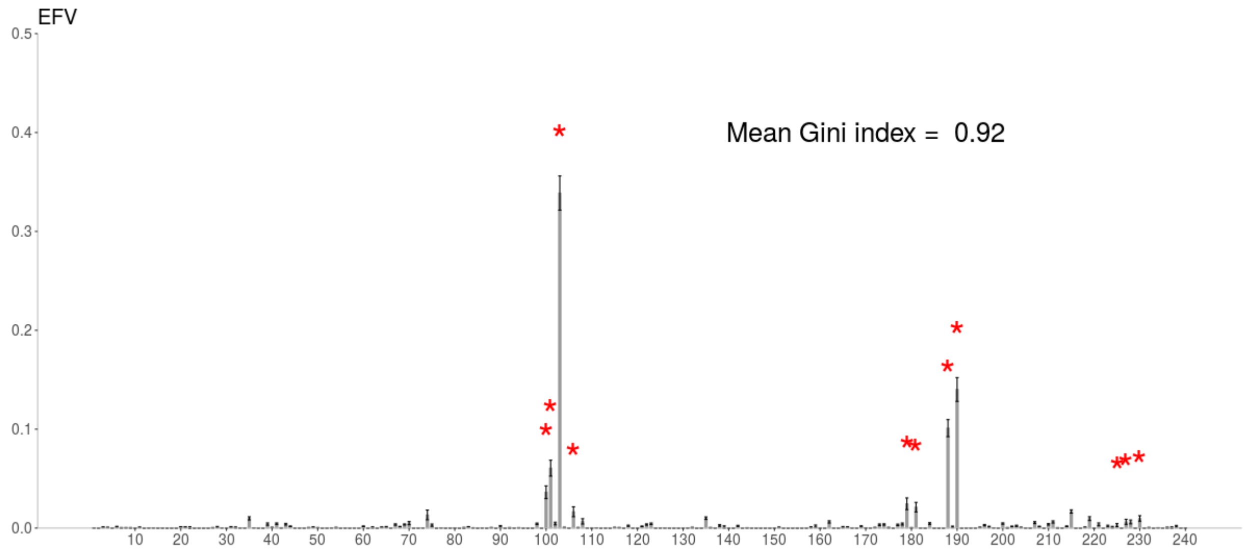

Fig. S31. RF relative importance of each protein position, averaged over 40 replicates, for EFV (reverse transcriptase inhibitor). Asterisks mark the major drug-related positions reported in the literature.

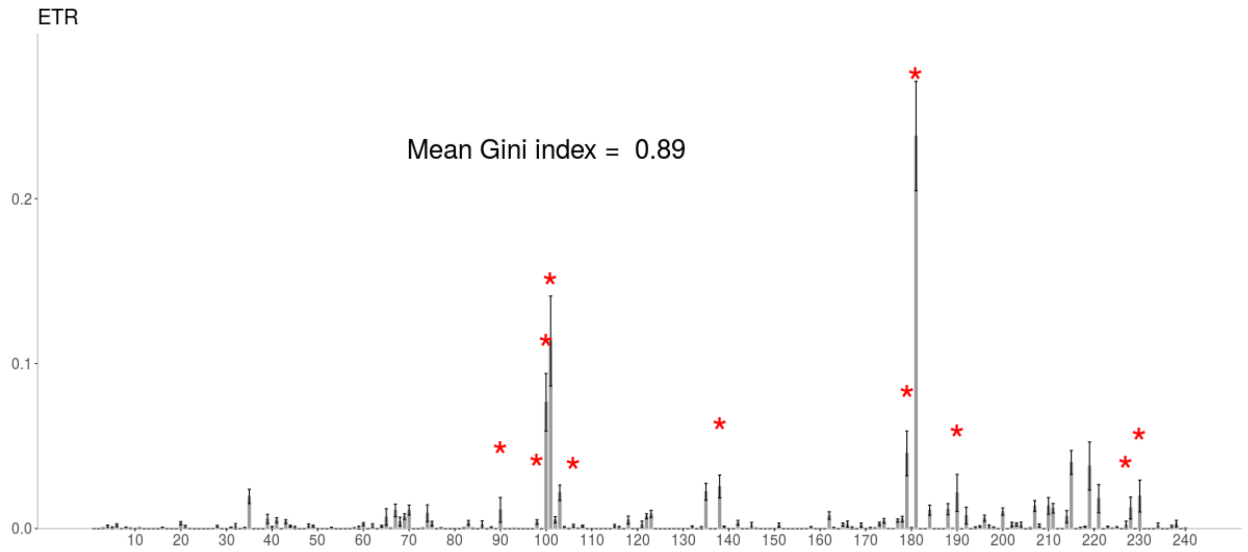

Fig. S32. RF relative importance of each protein position, averaged over 40 replicates, for ETR (reverse transcriptase inhibitor). Asterisks mark the major drug-related positions reported in the literature.

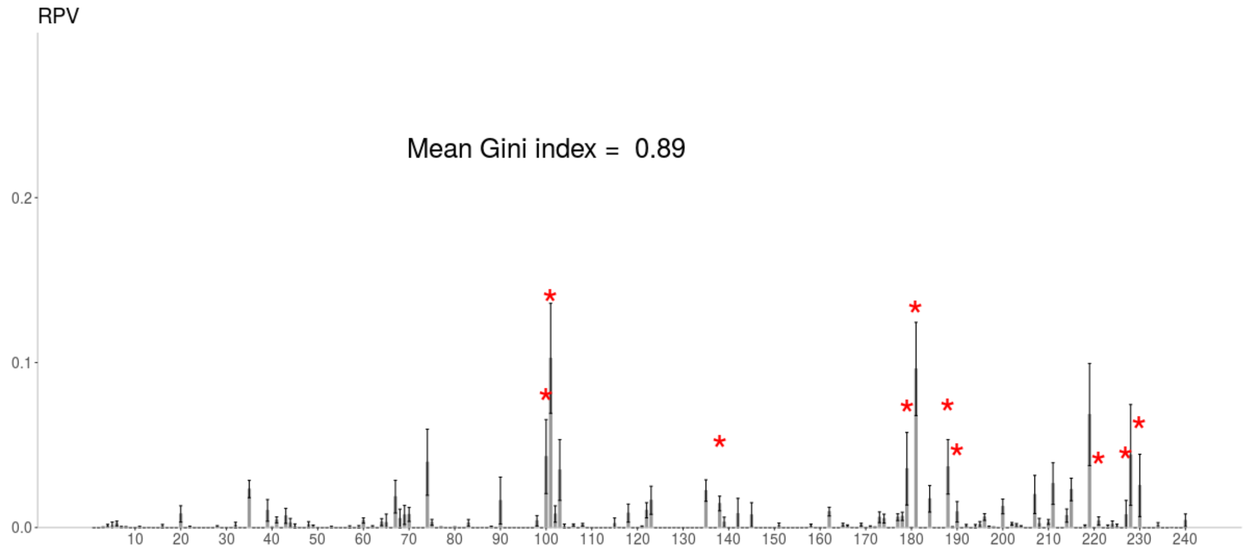

Fig. S33. RF relative importance of each protein position, averaged over 40 replicates, for RPV (reverse transcriptase inhibitor). Asterisks mark the major drug-related positions reported in the literature.

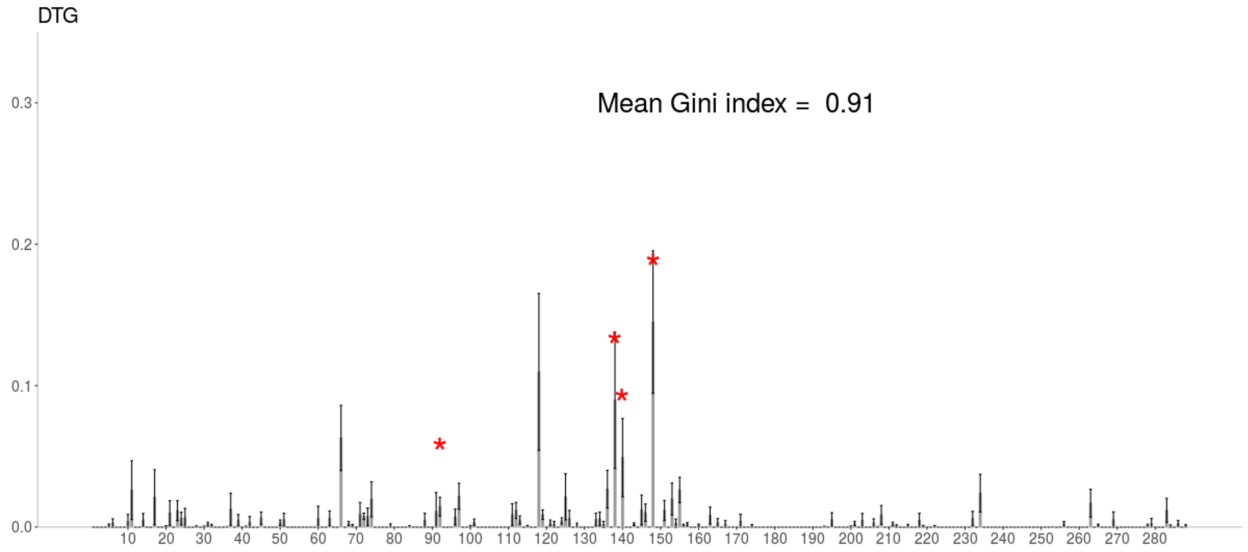

Fig. S34. RF relative importance of each protein position, averaged over 40 replicates, for DTG (integrase inhibitor). Asterisks mark the major drug-related positions reported in the literature.

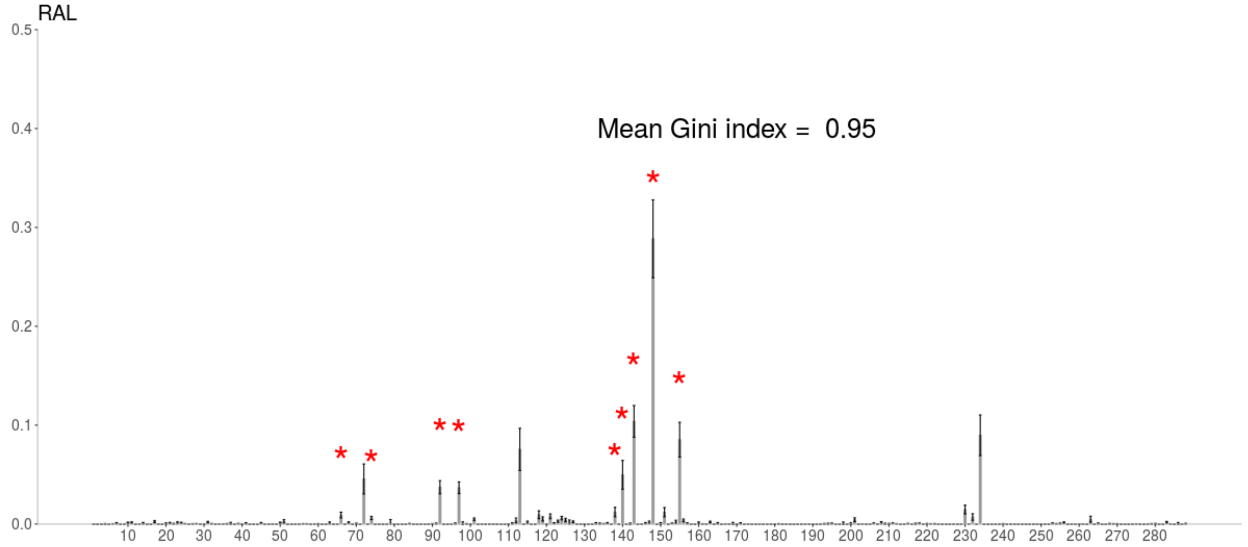

Fig. S35. RF relative importance of each protein position, averaged over 40 replicates, for RAL (integrase inhibitor). Asterisks mark the major drug-related positions reported in the literature.
