# Supplementary material for: HIV drug resistance prediction with weighted categorical kernel functions
Source: BMC Bioinformatics. 2019 Jul 30;20:410. doi: 10.1186/s12859-019-2991-2 (PMC6668108; doi:10.1186/s12859-019-2991-2)
Supplement: Supplementary file 3 — Table S1. Average NMSE of stacked methods. Table S2. NMSE Standard error of stacked methods (PDF 42 kb) [file 12859_2019_2991_MOESM3_ESM.pdf]

Table S1. Mean NMSE for all 21 analyzed drugs. wLIN, wRBF, wOV and wJAC stand for (individual) weighted Linear, RBF, Overlap and Jaccard models. sLIN, sRBF, sOV and sJAC correspond to their stacked counterparts.

	wLIN	sLIN	wRBF	sRBF	wOV	sOV	wJAC	sJAC
ATV	0.136	0.136	0.146	0.149	0.137	0.137	0.132	0.132
DRV	0.200	0.197	0.190	0.192	0.190	0.196	0.175	0.182
FPV	0.155	0.162	0.156	0.165	0.150	0.158	0.141	0.148
IDV	0.135	0.141	0.134	0.142	0.129	0.136	0.126	0.134
LPV	0.108	0.112	0.110	0.118	0.105	0.113	0.098	0.102
NFV	0.143	0.138	0.133	0.136	0.130	0.134	0.123	0.126
SQV	0.151	0.146	0.145	0.148	0.142	0.146	0.130	0.133
TPV	0.365	0.398	0.346	0.387	0.348	0.377	0.333	0.358
3TC	0.146	0.134	0.112	0.182	0.108	0.163	0.075	0.091
ABC	0.186	0.189	0.162	0.187	0.158	0.177	0.138	0.153
AZT	0.222	0.229	0.206	0.218	0.201	0.217	0.181	0.194
D4T	0.229	0.270	0.223	0.255	0.230	0.257	0.226	0.251
DDI	0.256	0.279	0.250	0.281	0.247	0.276	0.237	0.267
TDF	0.397	0.454	0.381	0.475	0.390	0.453	0.367	0.431
EFV	0.181	0.168	0.157	0.161	0.154	0.165	0.122	0.126
ETR	0.360	0.386	0.345	0.366	0.320	0.331	0.312	0.321
NVP	0.269	0.165	0.164	0.155	0.144	0.155	0.110	0.112
RPV	0.529	0.524	0.516	0.522	0.466	0.474	0.455	0.461
DTG	0.759	0.779	0.677	0.726	0.686	0.729	0.654	0.679
EVG	0.275	0.238	0.212	0.212	0.186	0.196	0.142	0.144
RAL	0.165	0.153	0.139	0.141	0.140	0.154	0.105	0.110

Table S2. NMSE standard error for all 21 analyzed drugs. Same abbreviations as in Table S1.

	wLIN	sLIN	wRBF	sRBF	wOV	sOV	wJAC	sJAC
ATV	0.013	0.016	0.014	0.017	0.014	0.014	0.014	0.015
DRV	0.018	0.029	0.018	0.024	0.018	0.024	0.018	0.017
FPV	0.011	0.012	0.010	0.013	0.009	0.010	0.009	0.009
IDV	0.009	0.010	0.008	0.008	0.008	0.007	0.008	0.008
LPV	0.010	0.013	0.013	0.016	0.011	0.012	0.009	0.011
NFV	0.009	0.009	0.009	0.009	0.007	0.008	0.006	0.008
SQV	0.009	0.012	0.008	0.011	0.008	0.009	0.008	0.008
TPV	0.038	0.047	0.040	0.050	0.043	0.048	0.040	0.044
3TC	0.022	0.026	0.021	0.038	0.020	0.043	0.013	0.016
ABC	0.016	0.019	0.016	0.020	0.014	0.015	0.015	0.015
AZT	0.017	0.022	0.017	0.021	0.018	0.021	0.012	0.016
D4T	0.028	0.032	0.027	0.030	0.027	0.035	0.027	0.033
DDI	0.023	0.029	0.023	0.034	0.022	0.022	0.020	0.025
TDF	0.041	0.053	0.039	0.100	0.039	0.065	0.039	0.065
EFV	0.016	0.017	0.013	0.017	0.014	0.017	0.012	0.013
ETR	0.053	0.055	0.052	0.061	0.042	0.045	0.047	0.047
NVP	0.023	0.021	0.019	0.020	0.017	0.017	0.011	0.012
RPV	0.132	0.130	0.127	0.147	0.106	0.114	0.096	0.106
DTG	0.159	0.175	0.129	0.166	0.128	0.130	0.136	0.166
EVG	0.046	0.048	0.036	0.044	0.036	0.039	0.032	0.033
RAL	0.040	0.040	0.033	0.035	0.037	0.041	0.029	0.032
