# Supplementary material for: HIV drug resistance prediction with weighted categorical kernel functions
Source: BMC Bioinformatics. 2019 Jul 30;20:410. doi: 10.1186/s12859-019-2991-2 (PMC6668108; doi:10.1186/s12859-019-2991-2)
Supplement: Supplementary file 4 — Figures S36-S54. Kernel PCAs for drugs ATV, DRV, IDV, LPV, NFV, TPV, SQV, 3TC, ABC, AZT, D4T, DDI, TDF, EFV, ETR, RPV, DTG, EVG and RAL (PDF 2075 kb) [file 12859_2019_2991_MOESM4_ESM.pdf]

ADDITIONAL FILE 3

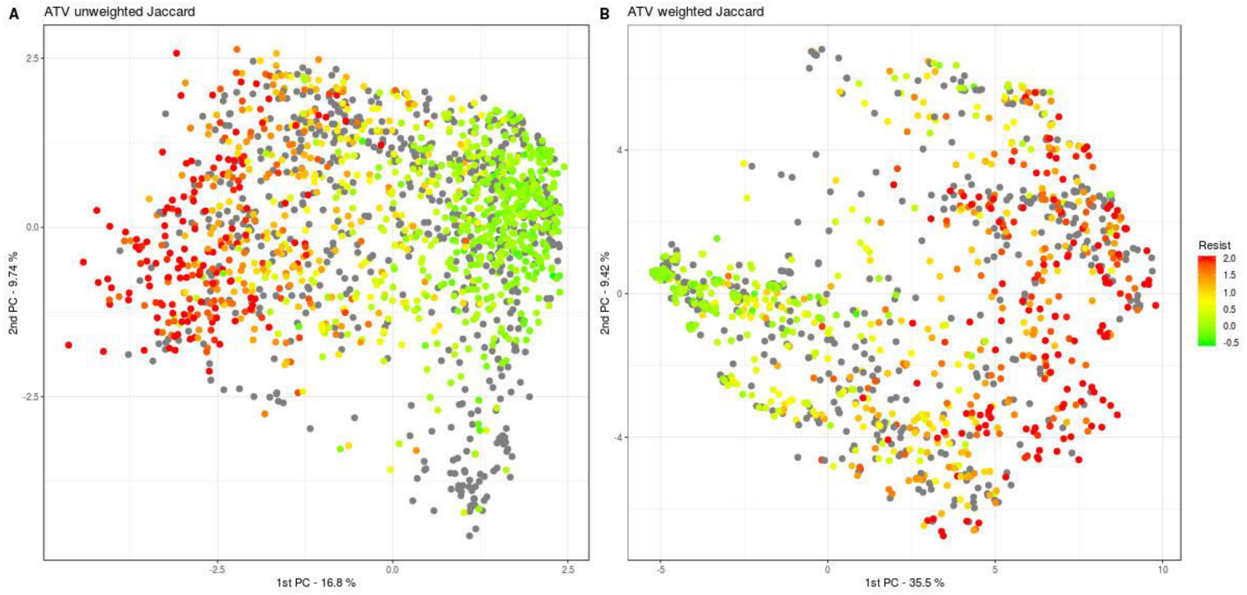

Fig. S36. The unweighted (A) and weighted (B) Jaccard Kernel PCA for ATV (protease inhibitor). Gray dots represent sequences with missing resistance value.

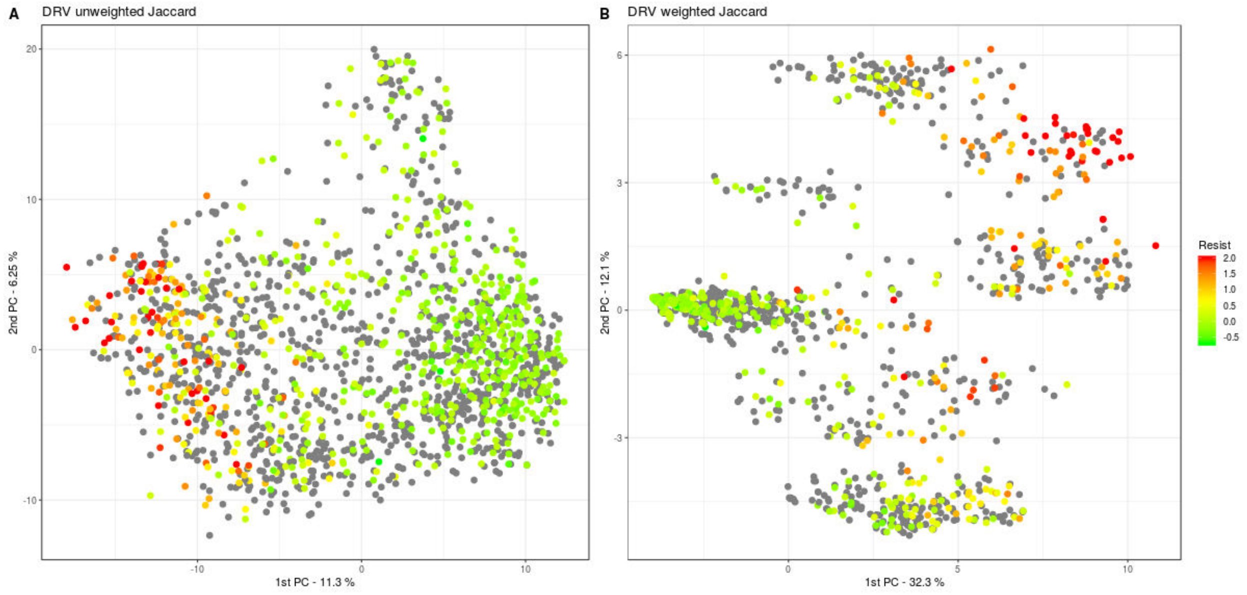

Fig. S37. The unweighted (A) and weighted (B) Jaccard Kernel PCA for DRV (protease inhibitor). Gray dots represent sequences with missing resistance value.

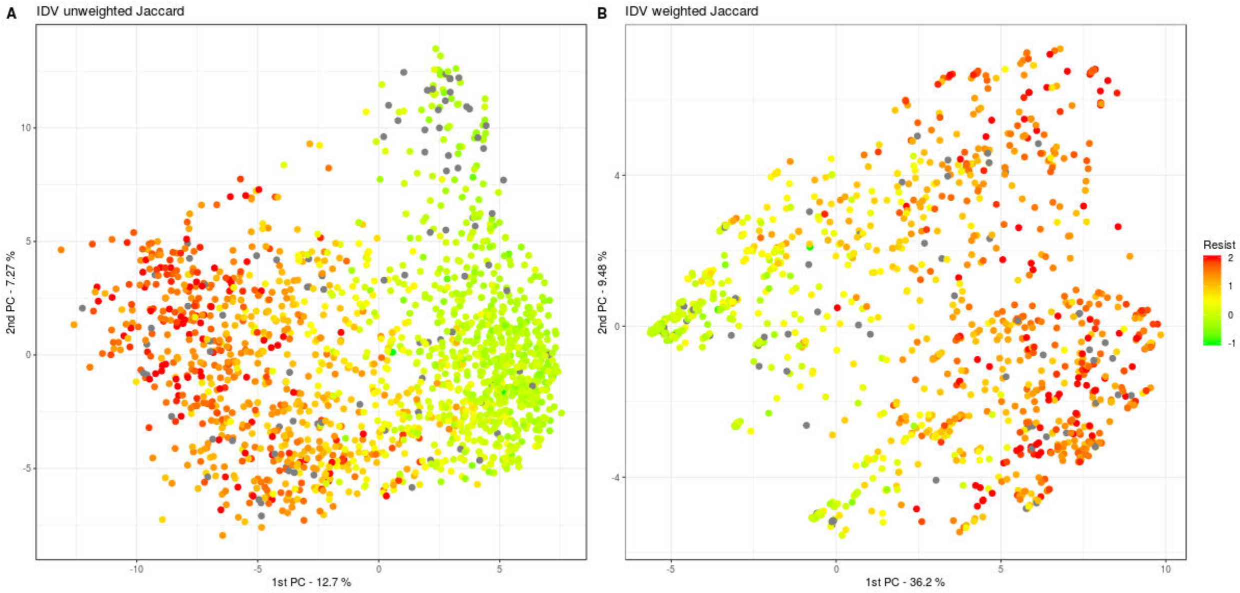

Fig. S38. The unweighted (A) and weighted (B) Jaccard Kernel PCA for IDV (protease inhibitor). Gray dots represent sequences with missing resistance value.

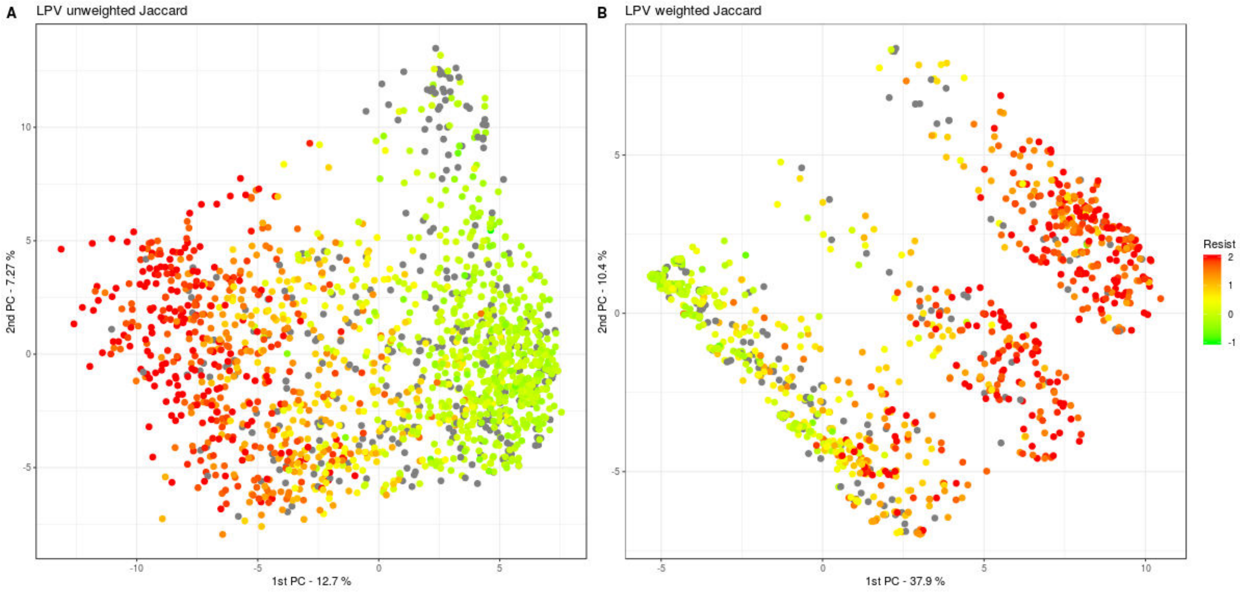

Fig. S39. The unweighted (A) and weighted (B) Jaccard Kernel PCA for LPV (protease inhibitor). Gray dots represent sequences with missing resistance value.

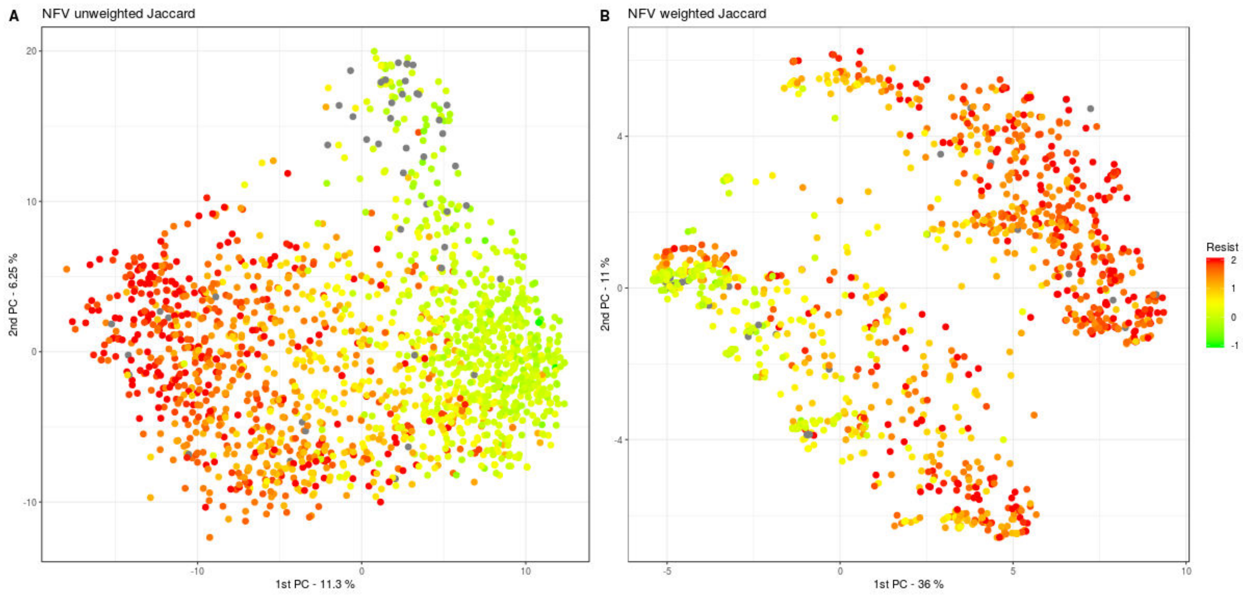

Fig. S40. The unweighted (A) and weighted (B) Jaccard Kernel PCA for NFV (protease inhibitor). Gray dots represent sequences with missing resistance value.

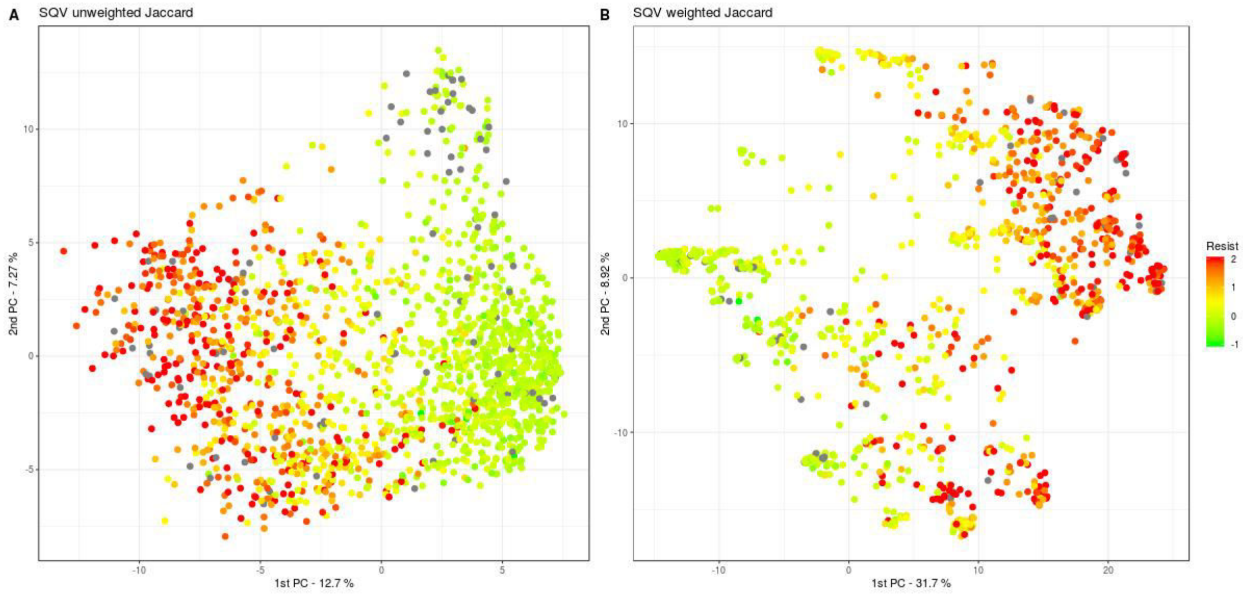

Fig. S41. The unweighted (A) and weighted (B) Jaccard Kernel PCA for SQV (protease inhibitor). Gray dots represent sequences with missing resistance value.

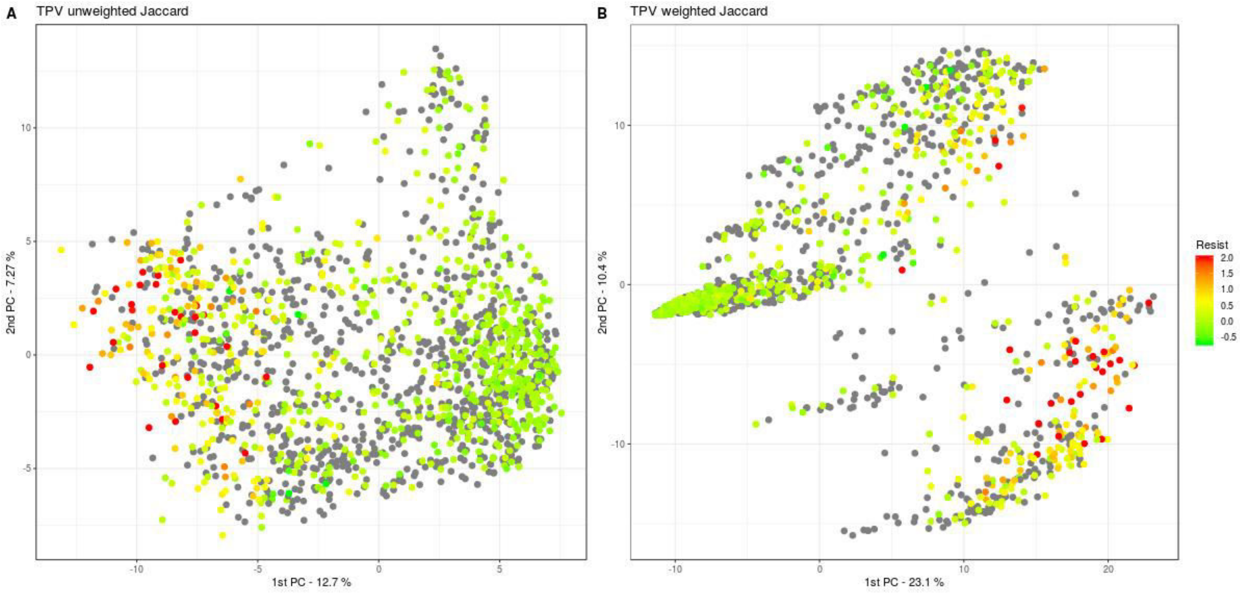

Fig. S42. The unweighted (A) and weighted (B) Jaccard Kernel PCA for TPV (protease inhibitor). Gray dots represent sequences with missing resistance value.

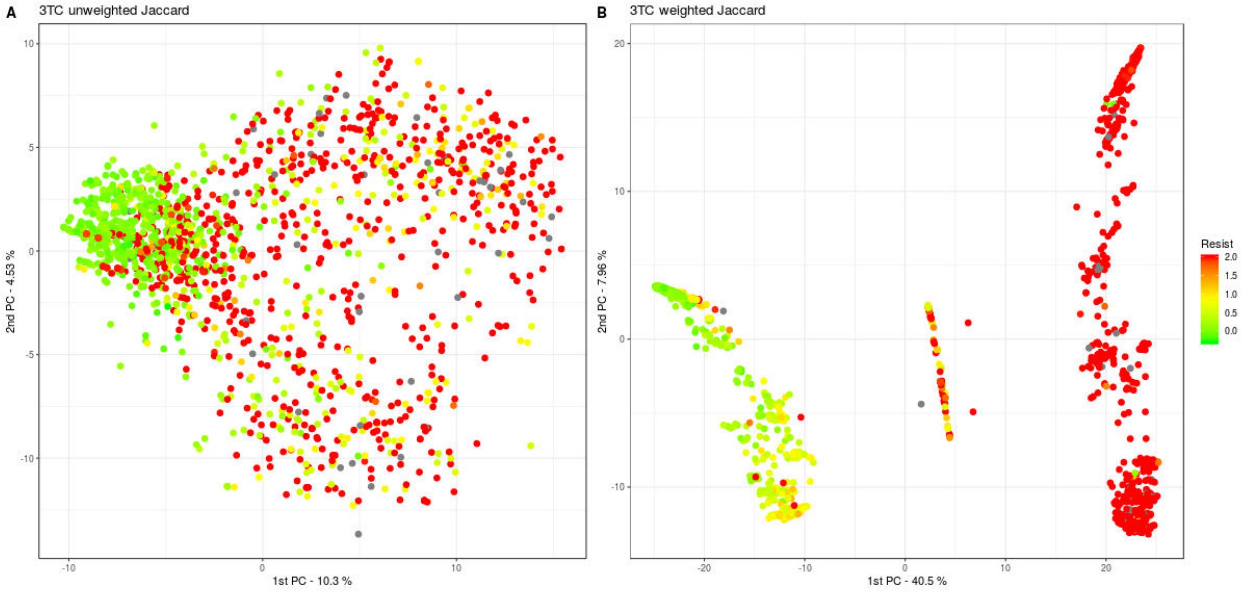

Fig. S43. The unweighted (A) and weighted (B) Jaccard Kernel PCA for 3TC (reverse transcriptase inhibitor). Gray dots represent sequences with missing resistance value.

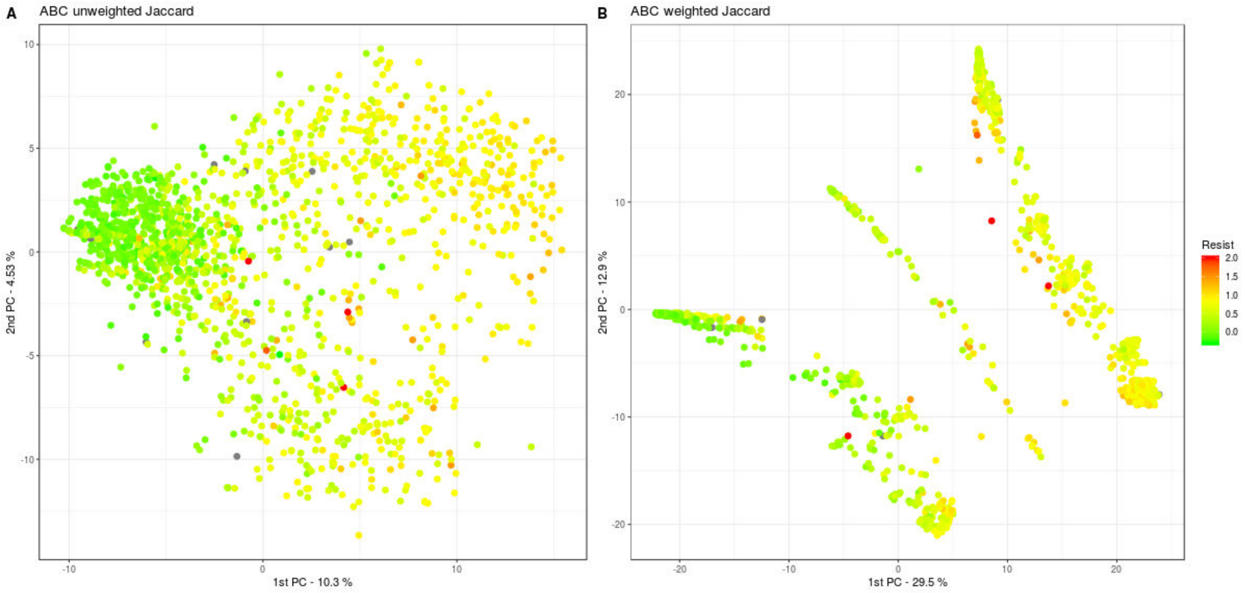

Fig. S44. The unweighted (A) and weighted (B) Jaccard Kernel PCA for ABC (reverse transcriptase inhibitor). Gray dots represent sequences with missing resistance value.

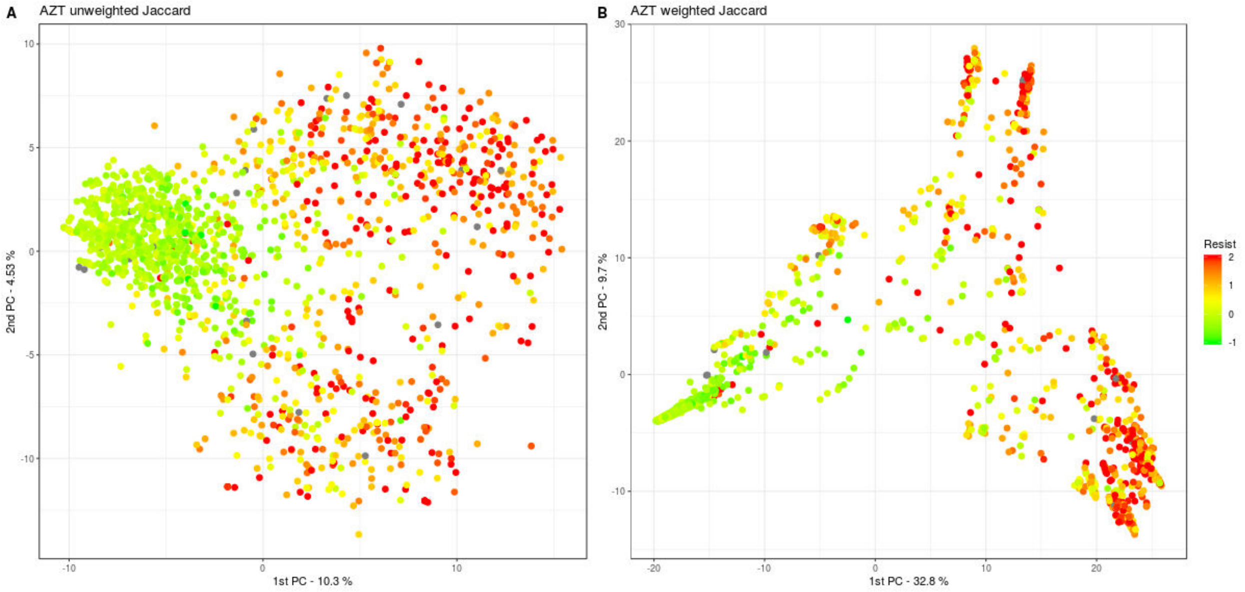

Fig. S45. The unweighted (A) and weighted (B) Jaccard Kernel PCA for AZT (reverse transcriptase inhibitor). Gray dots represent sequences with missing resistance value.

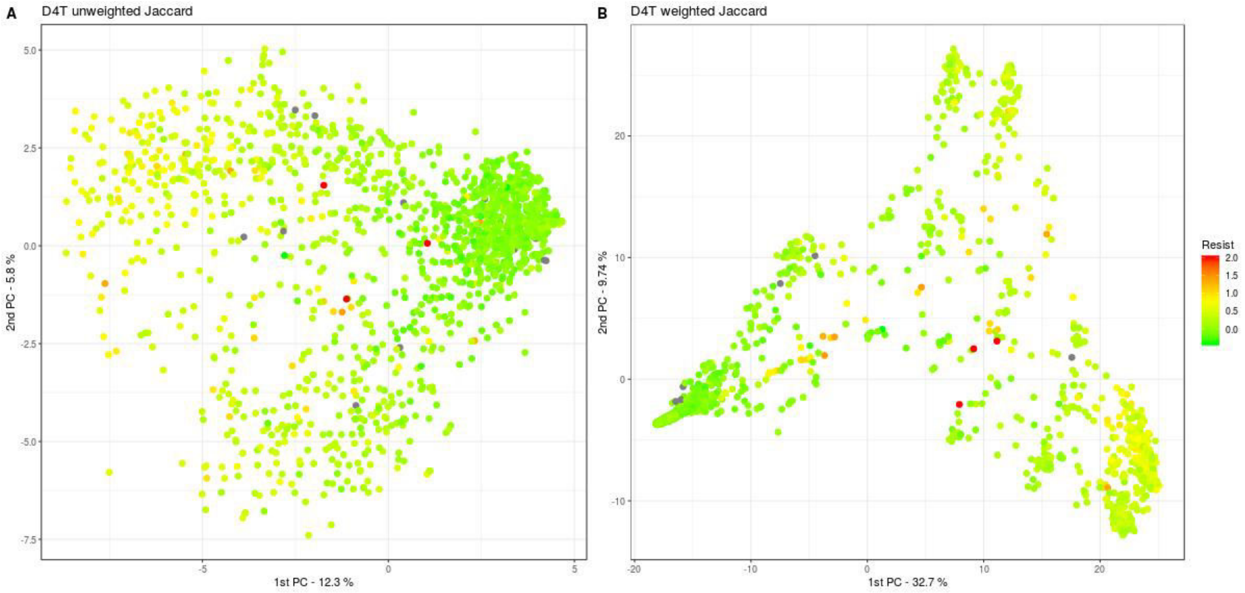

Fig. S46. The unweighted (A) and weighted (B) Jaccard Kernel PCA for D4T (reverse transcriptase inhibitor). Gray dots represent sequences with missing resistance value.

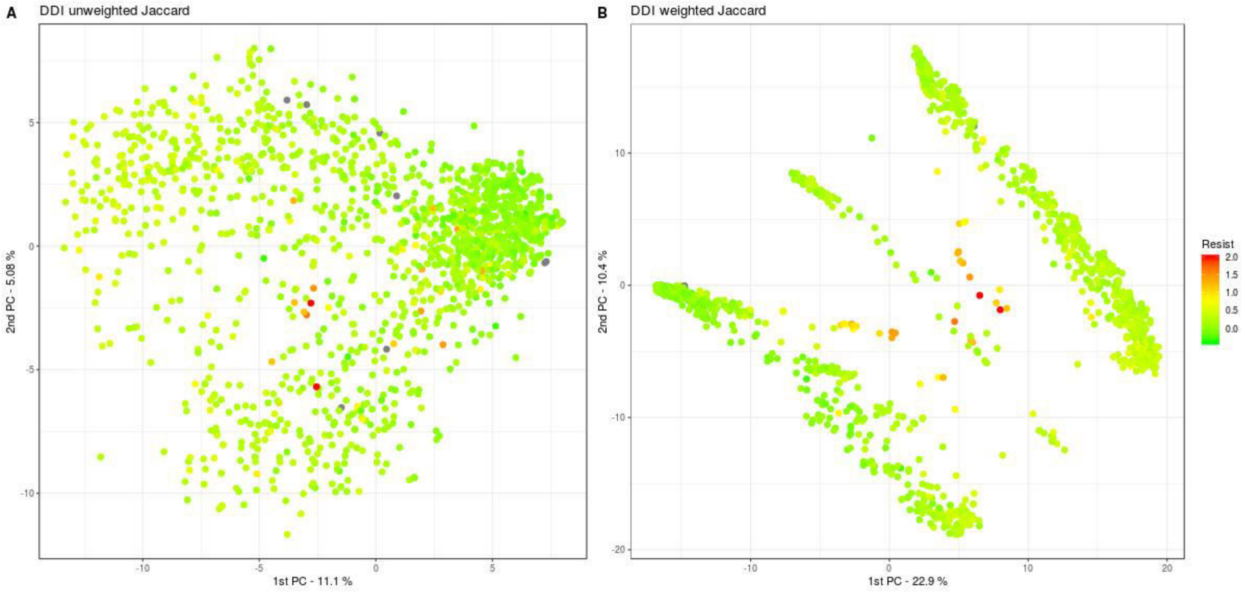

Fig. S47. The unweighted (A) and weighted (B) Jaccard Kernel PCA for DDI (reverse transcriptase inhibitor). Gray dots represent sequences with missing resistance value.

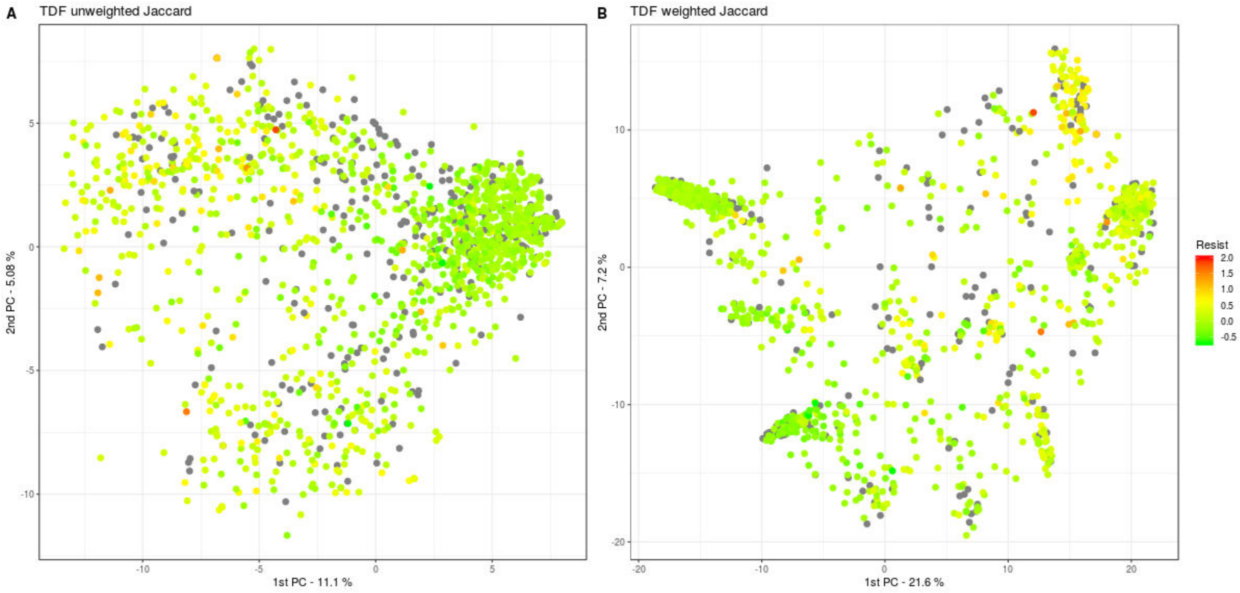

Fig. S48. The unweighted (A) and weighted (B) Jaccard Kernel PCA for TDF (reverse transcriptase inhibitor). Gray dots represent sequences with missing resistance value.

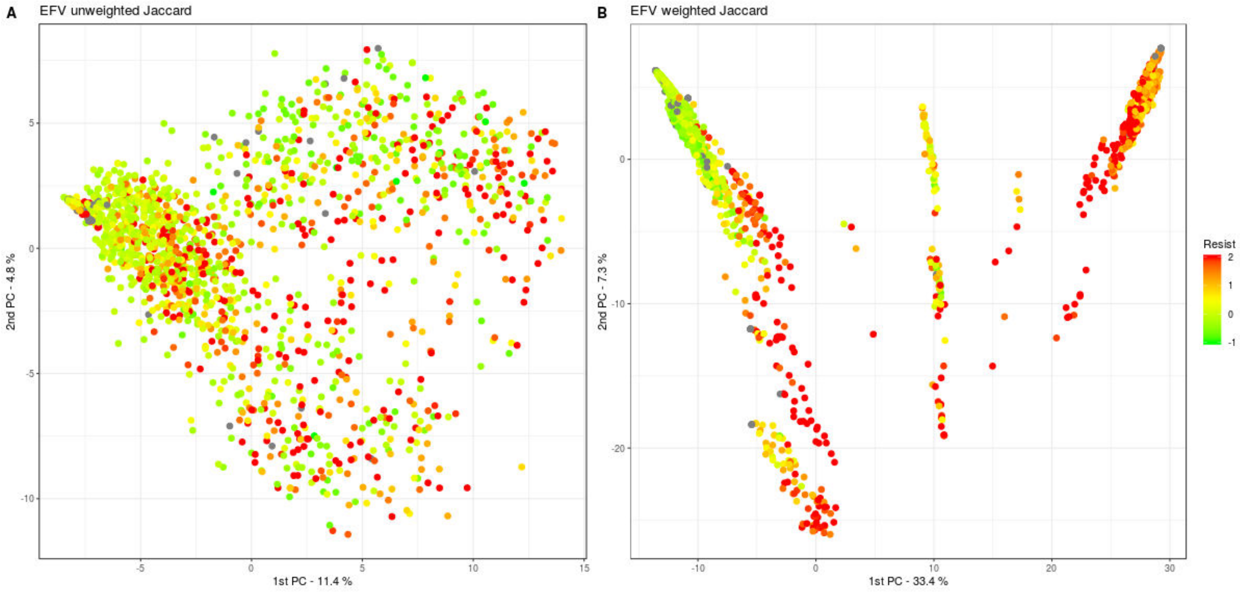

Fig. S49. The unweighted (A) and weighted (B) Jaccard Kernel PCA for EFV (reverse transcriptase inhibitor). Gray dots represent sequences with missing resistance value.

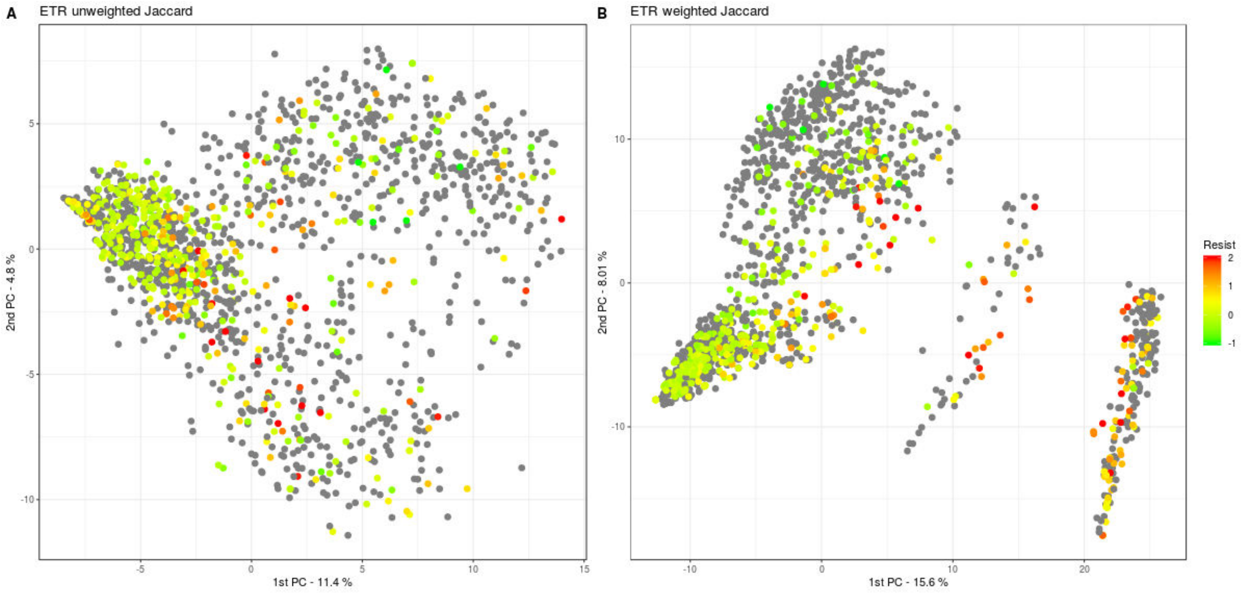

Fig. S50. The unweighted (A) and weighted (B) Jaccard Kernel PCA for ETR (reverse transcriptase inhibitor). Gray dots represent sequences with missing resistance value.

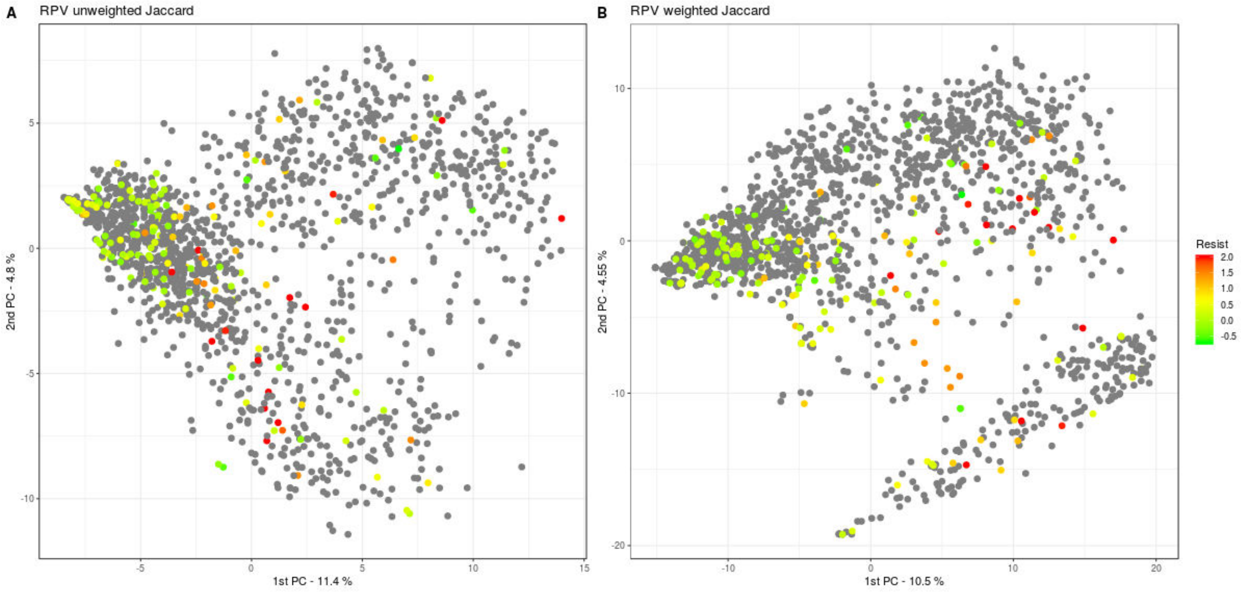

Fig. S51. The unweighted (A) and weighted (B) Jaccard Kernel PCA for RPV (reverse transcriptase inhibitor). Gray dots represent sequences with missing resistance value.

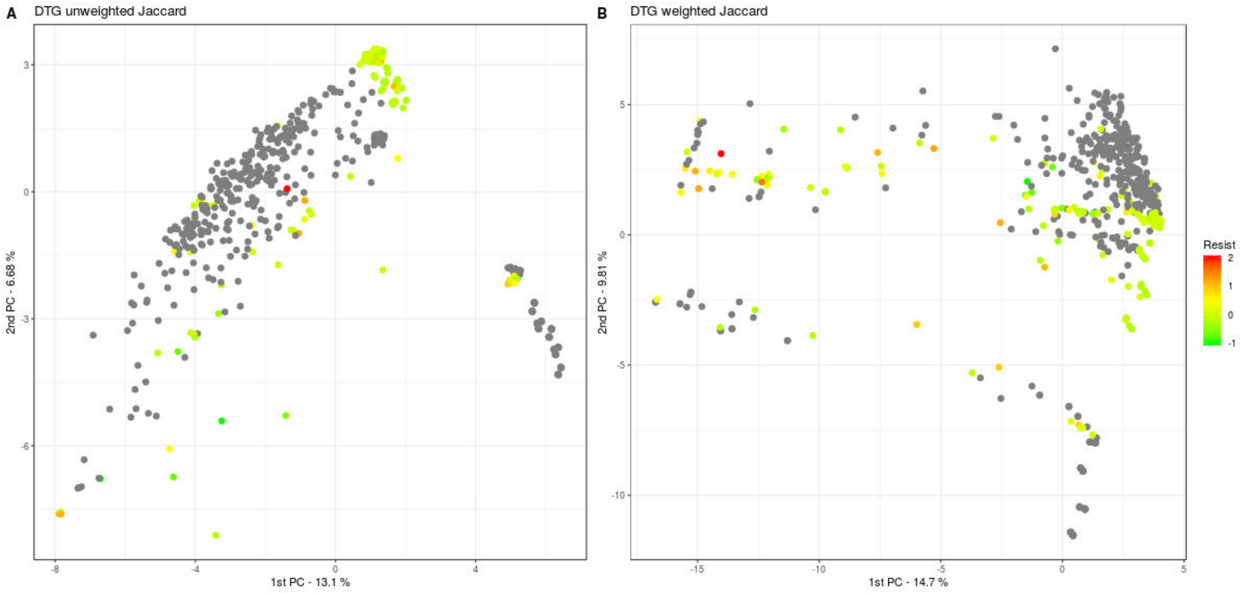

Fig. S52. The unweighted (A) and weighted (B) Jaccard Kernel PCA for DTG (integrase inhibitor). Gray dots represent sequences with missing resistance value.

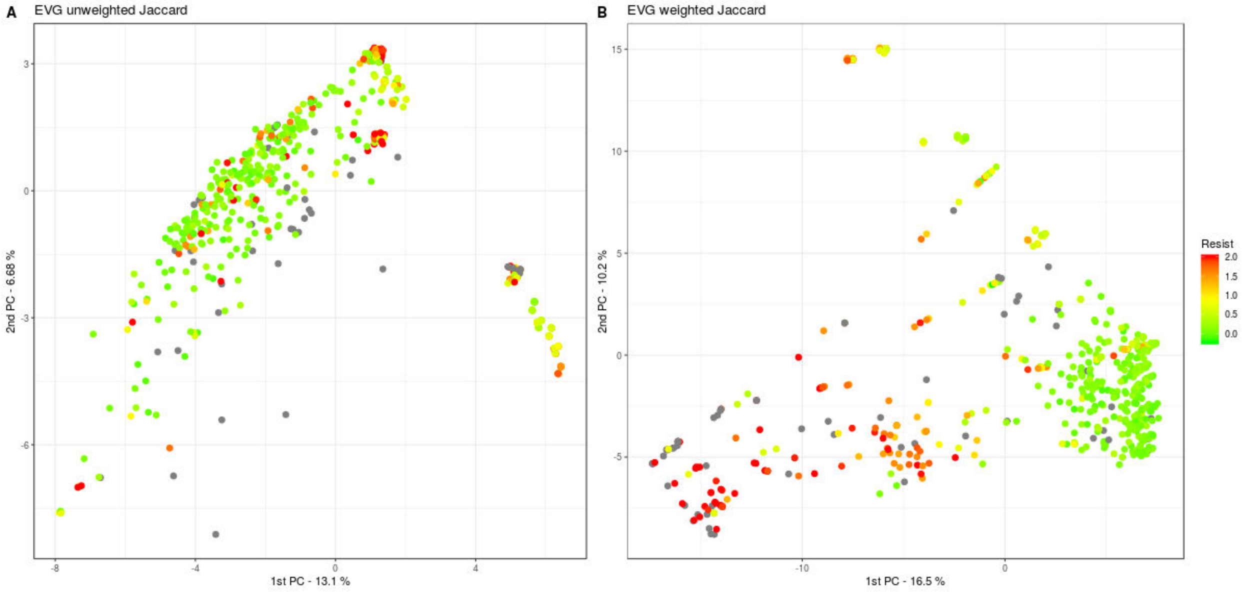

Fig. S53. The unweighted (A) and weighted (B) Jaccard Kernel PCA for EVG (integrase inhibitor). Gray dots represent sequences with missing resistance value.

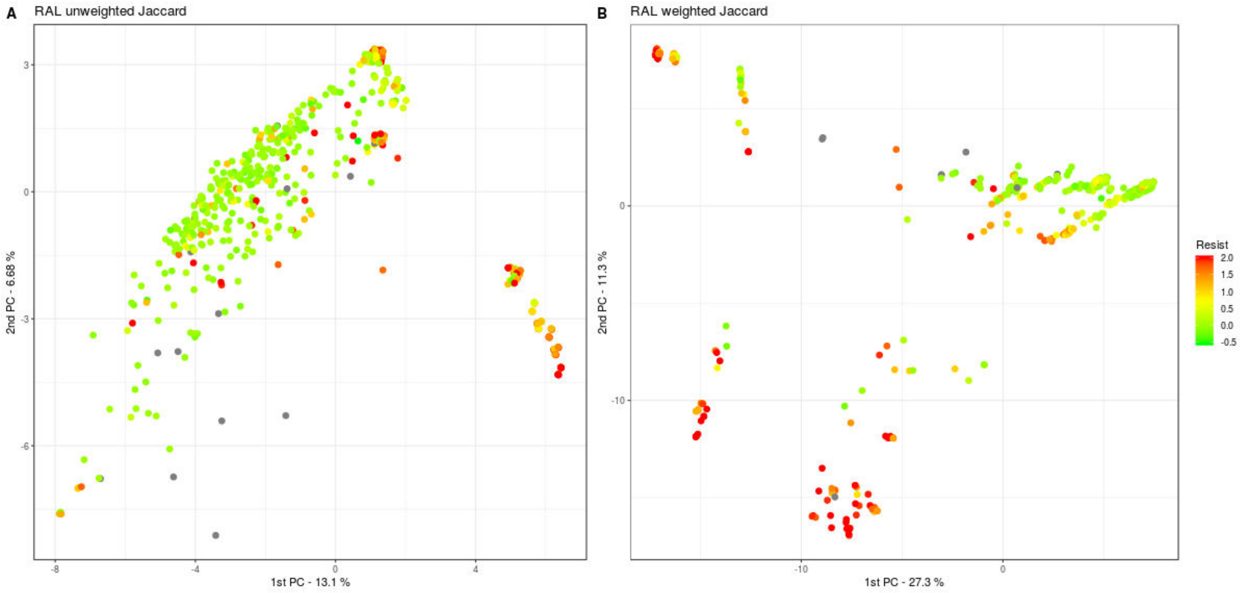

Fig. S54. The unweighted (A) and weighted (B) Jaccard Kernel PCA for RAL (integrase inhibitor). Gray dots represent sequences with missing resistance value.
